# A systematic comparison of triterpenoid biosynthetic enzymes for the production of oleanolic acid in *Saccharomyces cerevisiae*

**DOI:** 10.1371/journal.pone.0231980

**Published:** 2020-05-01

**Authors:** Matthew P. Dale, Tessa Moses, Emily J. Johnston, Susan J. Rosser

**Affiliations:** School of Biological Sciences, University of Edinburgh, Edinburgh, Scotland, United Kingdom; CNR, ITALY

## Abstract

Triterpenoids are high-value plant metabolites with numerous applications in medicine, agriculture, food, and home and personal care products. However, plants produce triterpenoids in low abundance, and their complex structures make their chemical synthesis prohibitively expensive and often impossible. As such, the yeast *Saccharomyces cerevisiae* has been explored as an alternative means of production. An important triterpenoid is oleanolic acid because it is the precursor to many bioactive triterpenoids of commercial interest, such as QS-21 which is being evaluated as a vaccine adjuvant in clinical trials against HIV and malaria. Oleanolic acid is derived from 2,3-oxidosqualene (natively produced by yeast) via a cyclisation and a multi-step oxidation reaction, catalysed by a β-amyrin synthase and a cytochrome P450 of the CYP716A subfamily, respectively. Although many homologues have been characterised, previous studies have used arbitrarily chosen β-amyrin synthases and CYP716As to produce oleanolic acid and its derivatives in yeast. This study presents the first comprehensive comparison of β-amyrin synthase and CYP716A enzyme activities in yeast. Strains expressing different homologues are compared for production, revealing 6.3- and 4.5-fold differences in β-amyrin and oleanolic acid productivities and varying CYP716A product profiles, which are important to consider when engineering strains for the production of bioactive oleanolic acid derivatives.

## 1. Introduction

Oleanolic acid is a plant specialized metabolite of considerable commercial interest that belongs to the oleanane class of triterpenoids. The oleanane scaffold is composed of 30 carbons arranged in a pentacyclic structure that is decorated with various functional groups to obtain vast structural and functional diversity. Several plant species produce oleanolic acid [1], which can accumulate as itself or as oxidized and glycosylated derivatives called saponins. Oleanolic acid is a particularly important triterpenoid, being the precursor to a range of bioactive triterpenoids such as the vaccine adjuvant QS-21, which is used in a vaccine against feline leukaemia virus and is being investigated in human vaccines against HIV and malaria [2]. Semi-synthetic derivatives of oleanolic acid have been investigated in the treatment of various diseases, including leukaemia, chronic kidney disease, type II diabetes, osteoporosis and Friedreich’s ataxia [3,4], while glycosylated derivatives of oleanolic acid have been shown to act as feeding deterrents to common crop pests [5].

**Fig 1:**
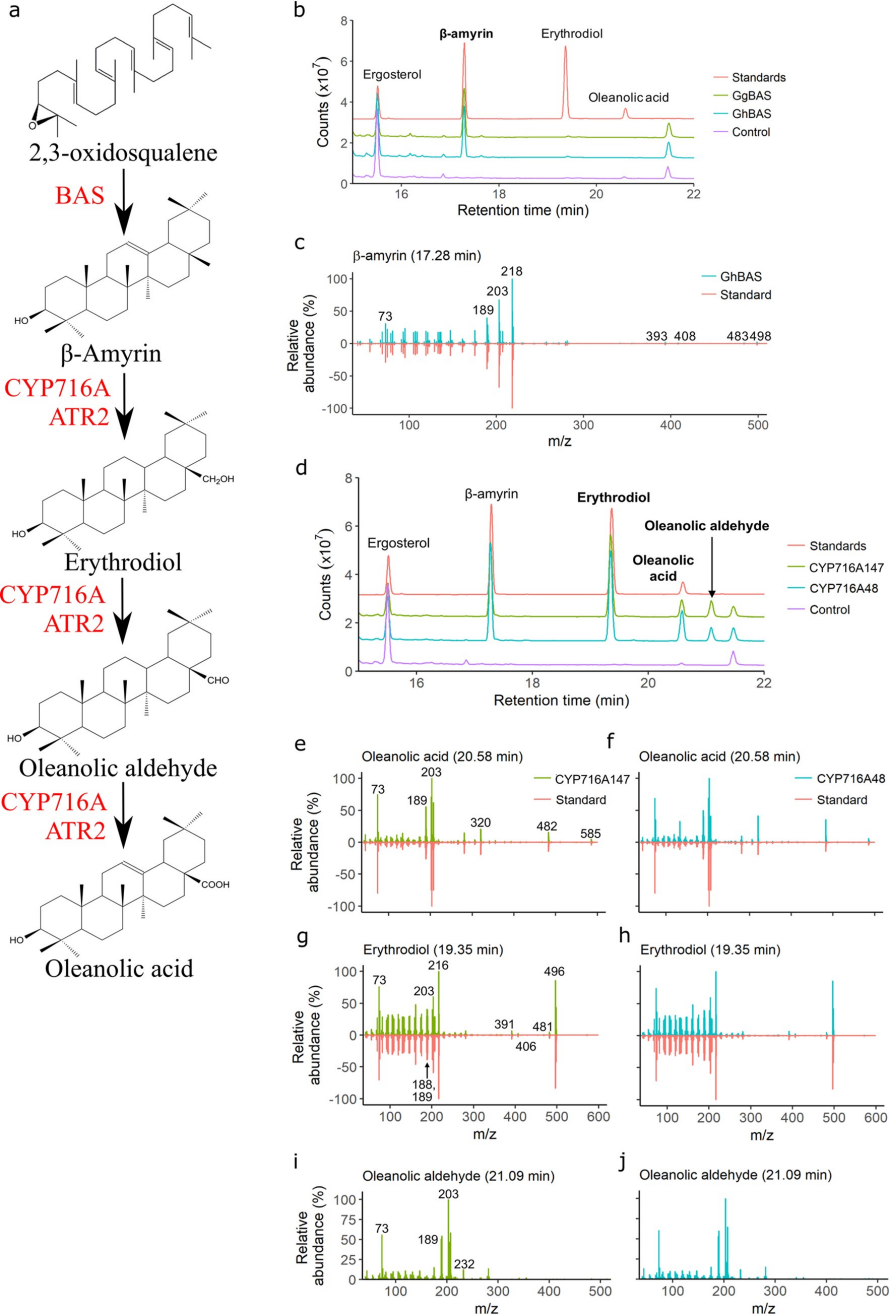
(a) Oleanolic acid biosynthesis pathway from 2,3-oxidosqualene. (b-j) GC/QTOF-MS confirms β-amyrin production by GhBAS, and oleanolic acid production by CYP716A147 and CYP716A48. (b, d) Total ion chromatograms of strains expressing GhBAS, CYP716A147 and CYP716A48, with control strains expressing GgBAS or empty vector, and a mixture of ergosterol, β-amyrin, erythrodiol and oleanolic acid standards at 50 μg/mL. (c) Mass spectra of the GhBAS β-amyrin peak matched to the standard. (e-h) Mass spectra of erythrodiol and oleanolic acid peaks from the CYP716A strains matched to standards. (i-j) Mass spectra of predicted oleanolic aldehyde peaks matched those previously reported.

As such, there is considerable interest in the production of oleanolic acid derivatives in industrially relevant quantities. Plants typically produce triterpenoids in low amounts in response to stress, and the complex stereochemistry of triterpenoids makes their chemical synthesis economically prohibitive and often impossible. Therefore, use of the yeast *Saccharomyces cerevisiae*, which is genetically tractable and widely used in industrial fermentations, has been explored for triterpenoid production [6–9]. Oleanolic acid biosynthesis occurs through the cyclisation of 2,3-oxidosqualene (natively produced by yeast) to produce β-amyrin, followed by the oxidation of carbon-28 (C-28) methyl group of β-amyrin to a carboxyl group. This oxidation reaction is predominantly catalysed by cytochrome P450 monooxygenases (P450s) of the CYP716A subfamily in conjunction with a cytochrome P450 reductase (CPR) partner, and proceeds through three steps, producing the intermediates erythrodiol and oleanolic aldehyde, which have a hydroxyl and aldehyde group at C-28, respectively (Fig 1A). Oleanolic acid can thus be produced in yeast by the co-expression of a β-amyrin synthase (BAS), a CYP716A and a CPR, with the expression of additional P450s and UDP-dependent glycosyltransferases allowing the production of bioactive oleanolic acid derivatives.

The choice of enzyme homologues can significantly impact productivity, as exemplified by β-amyrin titres of 19 and 36 mg/L when BASs from *Medicago truncatula* (*MtBAS*) and *Glycyrrhiza glabra* (*GgBAS*) were expressed in yeast [6]. In addition, titres of 107 and 1.9 mg/L β-amyrin have been reported for *GgBAS* and *PgBAS1* (from *Panax ginseng*) [7]. Despite these broad-ranging yields, no systematic comparison of BAS and CYP716A productivities in yeast have been reported. The advantage of such comparisons has previously been demonstrated for the CYP93E subfamily of P450s, which catalyse the three-step oxidation of the β-amyrin C-24 methyl group to a carboxyl group. A 52-fold difference in accumulation of 24-hydroxy-β-amyrin was reported between strains expressing eight different CYP93E variants [10]. As observed for the CYP93E subfamily, a comparative analysis of CYP716A enzymes would be particularly relevant due to the multi-step nature of the reaction catalysed, because the ratios of accumulated intermediates and product could differ between homologues. We therefore decided to conduct a comprehensive comparison of the productivities of BAS and CYP716A homologues in yeast, to identify the most productive variants for oleanolic acid production. Such variants could then be used to engineer high yields of oleanolic acid and its derivatives from yeast.

## 2. Materials and methods

### 2.1 Chemicals

Coprostan-3-ol, ethyl acetate and pyridine were purchased from Sigma-Aldrich. n-Hexane was obtained from Merck, N-methyl-N-trimethylsilyltrifluoroacetamide (MSTFA) from Thermo Scientific, and potassium hydroxide pellets from Scientific Laboratory Supplies. β-Amyrin, erythrodiol and oleanolic acid standards were purchased from Extrasynthese.

### 2.2 Media

The synthetic defined medium was composed of 6.9 g/L yeast nitrogen base without amino acids (Sigma) and 0.79 g/L complete supplement mixture or 0.77 g/L single drop-out of uracil (Formedium) with carbon source (either 2% ᴅ-glucose [Fisher Chemical], or 2% ᴅ-(+)-galactose [Acros Organics]).

### 2.3 Plasmid construction

All genes except *AsBAS* were codon-optimised for *S*. *cerevisiae*, synthesised by GeneArt and assembled into low-copy number plasmids expressing the *URA3* auxotrophic marker [11]. *AsBAS* could not be synthesised and the native *Avena strigosa* sequence was a gift from the Osbourn lab (John Innes Centre, UK).

Plasmid backbones were initially constructed using parts provided in the MoClo Yeast Toolkit (MoClo-YTK) [11]. Plasmids pCPS1ULA, pCP12ULA and pCP2EULA each consisted of a CEN6/ARS4 low-copy origin, URA3 selection marker, ColE1 origin and Amicillin resistance gene for maintanence in *E*. *coli*, as well as a cloning site for either a BAS, CYP716A or ATR2 transcriptional unit (consisting of a promoter, ORF and terminator), respectively. Plasmid pMGSEULS instead had an *E*. *coli* Spectinomycin resistance gene and a site into which three transcriptional units (BAS, CYP716A and ATR2) can be cloned to form an oleanolic acid biosynthetic pathway.

*BAS*, *CYP716A* and *ATR2* transcriptional units, and oleanolic pathways, were subsequently assembled into these vectors using the MoClo-YTK system [11]. *BAS* genes were cloned into pCPS1ULA with the P_GAL1_ promoter and *TDH1* terminator, *CYP716A* genes into pCP12ULA with P_GAL10_ and the *ENO2* terminator, and *ATR2* into pCP2EULA with P_GAL3_ and the *PGK1* terminator. The resulting transcriptional units were then cloned into pMGSEULS to form oleanolic acid pathways, in which *BAS*, *CYP716A* and *ATR2* are co-expressed.

### 2.4 Generation and culturing of strains

Yeast strains were generated by transforming plasmids into *S*. *cerevisiae* BY4741 (β-amyrin production strains by transforming pCPS1ULA-BAS plasmids, and oleanolic acid strains by transforming pMGSEULS-OA plasmids). Empty pCPS1ULA vector was used as a control. All strains were grown at 30°C in synthetic defined medium lacking uracil and containing either 2% ᴅ-glucose (for selection of transformants, maintenance of strains, or precultures prior to inoculation in culturing medium) or 2% ᴅ-(+)-galactose (for quantifying triterpenoid production). For production of triterpenoids, strains were precultured for 24 h, inoculated to a starting OD_600_ of 0.2 in galactose-containing medium, and grown for 96 h in glass vials.

### 2.5 Triterpenoid extraction

Cells from 1 mL culture were separated from media, resuspended in 500 μL lysis buffer (40% potassium hydroxide, 50% ethanol, 80 nM coprostan-3-ol) and boiled for 10 min. Lysates were cooled to ambient temperature and extracted twice with 500 μL n-hexane, and then twice with 500 μL ethyl acetate. Organic phases were pooled, 600 μL was evaporated to dryness in a GeneVac EZ-2 Elite evaporator, and trimethylsilylated with 50 μL pyridine: N-methyl-N-trimethylsilyltrifluoroacetamide (1:4) for gas chromatography quadrupole time-of-flight mass spectrometry (GC/QTOF-MS) analysis.

### 2.6 Gas chromatography–Mass spectrometry analysis

The triterpene profiles were analysed on an Agilent 7890B gas chromatogram (GC) coupled to an Agilent 7200B quadrupole time-of-flight mass spectrometer (QTOF-MS) with GERSTEL multipurpose sampler (MPS) robotics (Anatune). Trimethysilylated samples (1 μL) were injected at a split ratio of 10:1, with a split flow of 10 mL/min into a DB-5ms 40 m × 250 μm × 0.25 μm GC column (Agilent Technologies). Helium was used as the carrier gas at a flow rate of 1 mL/min, the inlet was set to 250°C and the GC oven was programmed to 100°C for 2 min, followed by ramping at 25°C/min to 300°C, where it was held for 25 min. The ion source were set to 230°C, 35 μA filament current, 70 eV electron energy and the mass range of 60–900 *m/z* was scanned at an acquisition rate of 4 spectra/s with a solvent delay of 13 min. Total ion chromatograms and mass spectra were analysed using the Agilent MassHunter Qualitative Analysis B.07.00 software, and peak areas were calculated using the Agile 2 integrator method. GC/Q-TOF MS peak areas were used to obtain standard curves for β-amyrin, erythrodiol and oleanolic acid. The concentration range of 0 to 120 μg/mL, with increments of 10 μg/mL, was used and the linear region of the curves allowed quantifying concentration of the triterpenes.

## 3. Results

### 3.1 Characterization of new BAS and CYP716A enzymes

We first identified new BAS and CYP716A homologues. BLASTp searches on NCBI using GgBAS from *G*. *glabra* [12] and CYP716A12 from *M*. *truncatula* [13] as queries identified hits with > 70% amino acid sequence identity. GhBAS from *Gossypium hirsutum* (cotton), CYP716A48 from *Olea europaea* (olive) and CYP716A147 from *Theobroma cacao* (cocoa tree) were selected for further analysis (S1 Table). While *G*. *hirsutum* and *T*. *cacao* produce triterpenoids [14,15], genes encoding oleanane biosynthetic enzymes have not been previously characterised. Similarly, *O*. *europaea* accumulates oleanolic acid [1], but a P450 involved in its biosynthesis has not yet been described.

*In silico* analysis of the GhBAS sequence (S1 Fig) identified the highly conserved DCTAE motif involved in initiation of the cyclization reaction [16,17], the MWCYCR motif known to confer product specificity [18], and the QW motifs that are speculated to be involved in BAS protein stability [19–21]. Sequence alignment of CYP716A147 and CYP716A48 with functionally characterised CYP716A proteins (S2 Fig) identified the proton transfer groove, ExxR motif, PERF motif and haem-binding loop, along with the N-terminal transmembrane anchor.

The selected genes were functionally characterised in yeast. *GhBAS* was expressed alone, while *CYP716A48* and *CYP716A147* were co-expressed with *GgBAS* and *ATR2*, a CPR from *Arabidopsis thaliana*. While yeast encodes its own native CPR (*NCP1*), it is often unable to support the catalytic activity of heterologously expressed plant P450s [22]. As such, a plant CPR gene is usually co-expressed with plant P450s, typically one of the *A*. *thaliana* CPRs, *ATR1* or *ATR2* [7,8,23], of which *ATR2* has been reported to have a higher catalytic activity [24,25].

Compared to a strain carrying an empty vector (MD-N1), the strain expressing *GhBAS* produced β-amyrin, with an identical retention time and fragmentation pattern as an authentic standard (Fig 1B and 1C). Similarly, peaks with retention times and fragmentation patterns matching standards of erythrodiol and oleanolic acid were observed from both CYP716A-expressing strains (Fig 1D–1H). An additional peak not present in MD-N1 was observed in these strains (Fig 1D), with mass spectra matching that reported for an oleanolic aldehyde standard (Fig 1I and 1J) [26]. Thus, GhBAS is a monofunctional BAS that cyclises 2,3-oxidosqualene to β-amyrin, while CYP716A147 and CYP716A48 are C-28 oxidases that oxidise β-amyrin to oleanolic acid via erythrodiol and oleanolic aldehyde intermediates.

### 3.2 Strains expressing AaBAS or CqBAS1 produce the highest titre of β-amyrin

We screened the amount of β-amyrin produced in yeast by 12 BAS proteins from different plant species (S1 Table). These included the BAS variants most commonly used in engineering studies (GgBAS, MtBAS and PgBAS1), as well as AsBAS, BvBAS, AaBAS, CqBAS1, EtBAS, LjBAS, PtBAS, SlBAS and the newly characterised GhBAS.

**Fig 2:**
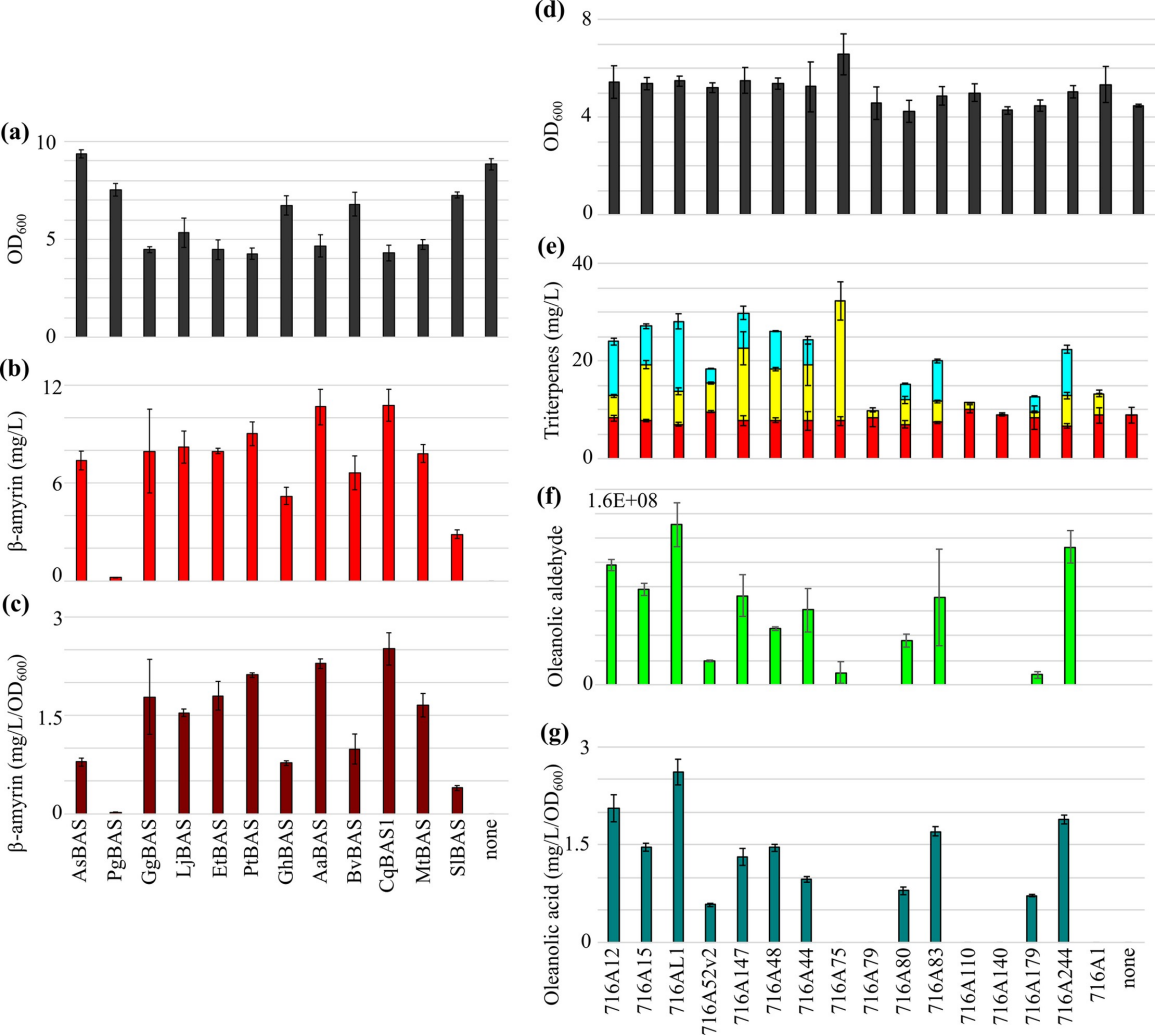
Comparison of BAS (a-c) and CYP716A (d-g) proteins for β-amyrin and oleanolic acid production. (a, d) OD_600_ at end of culture (grey). (b, e) Titres (mg/L) of β-amyrin (red), erythrodiol (yellow) and oleanolic acid (cyan). (f) Oleanolic aldehyde peak areas obtained from GC/QTOF-MS analysis. (c, g) β-Amyrin (dark red) and oleanolic acid (dark cyan) productivities (mg/L/OD_600_). None, empty vector strain MD-N1 in a-c and strain expressing *AaBAS* only in d-g. Mean ± SD for n = 3 is presented.

All strains except that expressing *AsBAS* displayed reduced growth compared to the control strain MD-N1 (Fig 2A). Furthermore, all strains except that expressing *PgBAS* produced quantifiable titres of β-amyrin (> 1.7 mg/L), with 3.7- and 6.3-fold differences in titre (mg/L) and productivity (mg/L/OD_600_), respectively, between the most and least productive strains in our study (Fig 2B and 2C). Overall, the highest titre of 10.8 ± 1.0 mg/L β-amyrin was produced by the strains expressing *AaBAS* or *CqBAS1*. The *PtBAS*, *LjBAS* and *EtBAS* strains closely followed, with β-amyrin titres of 9.0 ± 0.7, 8.2 ± 1.0 and 8.0 ± 0.2 mg/L, respectively. The *SlBAS* strain produced the lowest titre of 2.9 ± 0.3 mg/L β-amyrin.

Interestingly, in addition to β-amyrin, a second peak was observed in the total ion chromatogram of the BvBAS strain (S3A Fig). This peak appeared to be a combination of two peaks, suggesting the presence of two additional compounds, and was unique to this strain, not appearing in the other BAS strains or the MD-N1 control strain. Comparison of its mass spectrum with that of an authentic lupeol standard indicated that one of the additional compounds contained within the peak most likely corresponds to this triterpene (S3B Fig).

### 3.3 Strain expressing CYP716AL1 produces the highest titre of oleanolic acid

We next compared the activities of 16 CYP716A family proteins from a range of plant species (S1 Table) for their ability to oxidise β-amyrin to oleanolic acid. These included CYP716A12, which is commonly used in engineering studies, the newly identified CYP716A147 and CYP716A48, as well as CYP716A15, CYP716AL1, CYP716A52v2, CYP716A44, CYP716A75, CYP716A79, CYP716A80, CYP716A83, CYP716A110, CYP716A140, CYP716A179, CYP716A244 and CYP716A1.

All strains showed comparable growth to the control strain expressing *AaBAS* alone (Fig 2D) but displayed marked differences in triterpene production (Fig 2E), with oleanolic acid titre (mg/L) and productivity (mg/L/OD_600_) differing 4.8- and 4.5-fold, respectively, between strains (Fig 2E). Oxidation of β-amyrin was observed in all strains except that expressing *CYP716A140*, with oleanolic acid produced in 11 of the 16 strains (cyan bars, Fig 2E). The highest and lowest titres of 14.3 ± 1.6 and 3.0 ± 0.0 mg/L oleanolic acid were produced by strains expressing *CYP716AL1* and *CYP716A52v2*, respectively. CYP716AL1 also showed the highest oleanolic acid productivity (2.6 mg/L/OD_600_) and CYP716A52v2 the least (0.6 mg/L/OD_600_) (Fig 2G). Notably, all strains (except that expressing *CYP716A140*) accumulated varying amounts of erythrodiol (yellow bars, Fig 2E) and oleanolic aldehyde (green bars, Fig 2F; standard unavailable for quantification). Interestingly, CYP716A140 has previously been shown to oxidise β-amyrin to oleanolic acid, in a study using the *M*. *truncatula* CPR *MTR1* and a non-codon optimised CYP716A140 sequence [27]. These variances in the expression assay may explain the differences in activity observed for CYP716A140 in our study. The *CYP716A75* strain produced the highest erythrodiol titre (24.6 ± 3.9 mg/L), with trace amounts of oleanolic aldehyde and no oleanolic acid. Strains expressing *CYP716A79*, *CYP716A110* and *CYP716A1* accumulated 1.2 ± 0.1, 1.2 ± 0.1 and 4.5 ± 0.7 mg/L erythrodiol, respectively, with no oleanolic aldehyde or acid.

### 3.4 Combining optimal enzymes to boost oleanolic acid production

**Fig 3:**
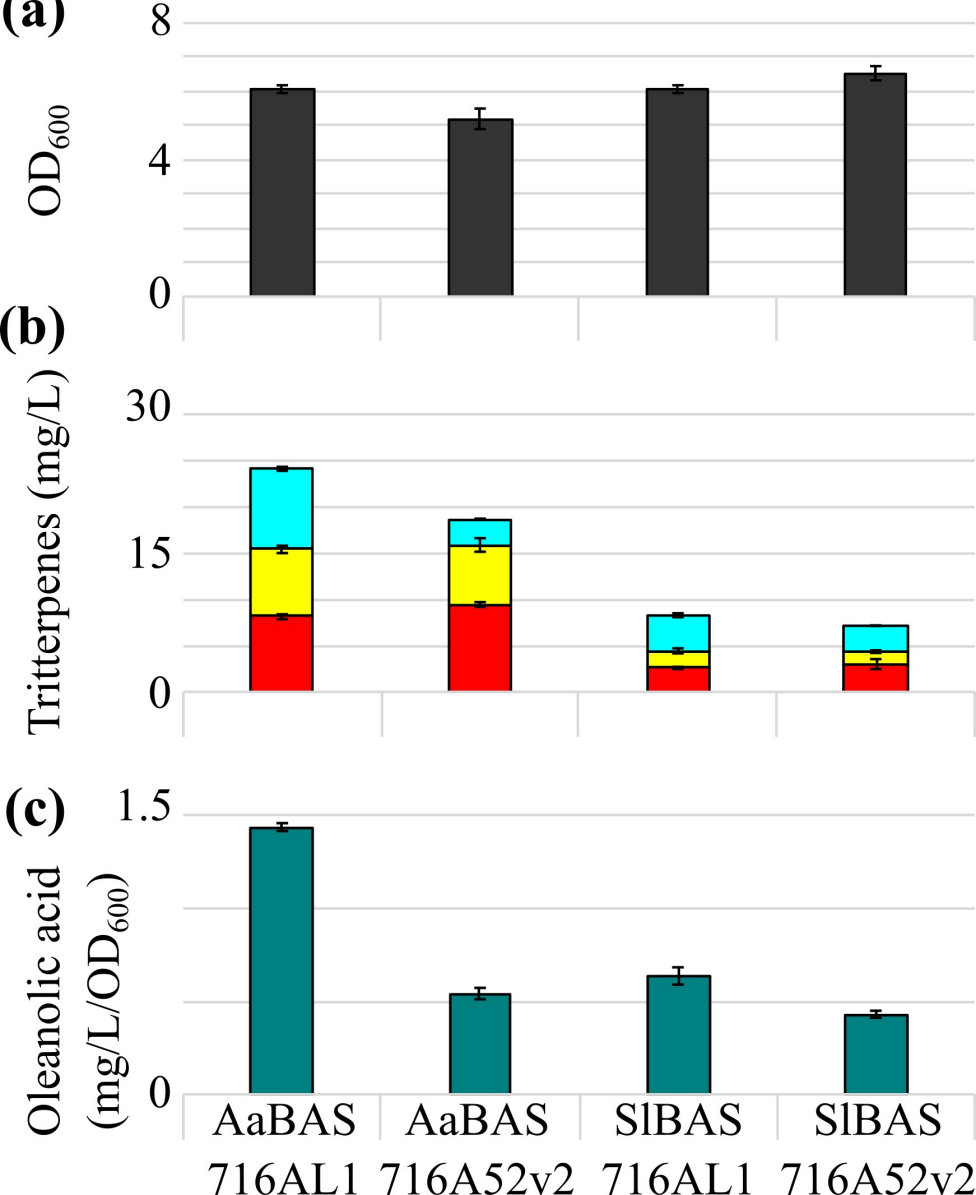
Triterpene production correlates to the activity of the BAS and CYP716A. (a) OD_600_ at end of culture (grey). (b) Titres (mg/L) of β-amyrin (red), erythrodiol (yellow) and oleanolic acid (cyan). (c) Oleanolic acid productivities (mg/L/OD_600_, dark cyan). Mean ± SD for n = 3 is presented.

To assess the impact of enzyme variant selection on oleanolic acid titres in yeast, we generated strains expressing *SlBAS* (the lowest β-amyrin titre) with *CYP716AL1* (the highest oleanolic acid titre) or *CYP716A52v2* (the lowest oleanolic acid titre) genes. Growth and oleanolic acid production was compared to strains expressing *AaBAS* (the highest β-amyrin titre) with *CYP716AL1* or *CYP716A52v2*. All strains showed comparable growth after 96 h of culturing (Fig 3A). The expression of *SlBAS* with *CYP716AL1* or *CYP716A52v2* resulted in 3.9 ± 0.2 and 2.8 ± 0.0 mg/L oleanolic acid, respectively (Fig 3B). The combination of *AaBAS* with *CYP716AL1* showed the highest titre (8.5 ± 0.2 mg/L) and productivity (1.40 ± 0.0 mg/L/OD_600_) of oleanolic acid (Fig 3B and 3C).

## 4. Discussion

In this study, a comprehensive comparison of 12 BAS and 16 CYP716A variants for the production of oleanolic acid in yeast was performed. These included enzymes commonly used in engineering studies (GgBAS, MtBAS, PgBAS1 and CYP716A12), as well as one BAS and two CYP716As newly characterised in this study.

These enzymes displayed a range of activities and product specificities, and impacted growth rates to different extents, when expressed in yeast. Notably, AaBAS and CqBAS1 produced higher titres of β-amyrin (10.8 mg/L), and CYP716AL1 produced higher titres of oleanolic acid (14.3 mg/L), than the BAS and CYP716A enzymes commonly used in engineering studies (GgBAS, MtBAS, PgBAS1 and CYP716A12). In addition, CYP716A75 predominantly produced the triterpenoid erythrodiol, suggesting an inherent ability of CYP716A75 to mainly catalyse the hydroxylation of β-amyrin to erythrodiol, as previously reported [28]. By contrast, CYP716AL1 and CYP716A12 produced substantially less erythrodiol, favouring the production of oleanolic acid. The use of P450s with different product specificities, such as those characterised here, will enable the production of a range of triterpenoids with specific modifications to the triterpene scaffold.

Interestingly, the strain expressing *AsBAS* was the only strain to show similar growth to the control strain MD-N1 carrying an empty vector. All other BAS strains displayed reduced growth compared to MD-N1. While experiments were not undertaken to determine the cause of this, it is notable that *AsBAS* was the only *BAS* gene that wasn’t codon-optimised for expression in yeast, which could have led to lower production of the AsBAS enzyme compared to the other BASs, possibly resulting in reduced burden on the cells. Protein sequence alignment also showed that AsBAS differed significantly in sequence to the other BAS proteins, having 47–50% sequence identity to them; by contrast, the other BAS proteins had 69–93% sequence identity to one another.

While the expression of *BAS* genes reduced growth by differing amounts, the additional expression of *CYP716A* and *ATR2* genes appeared to have much less impact on cell growth, with the growth of oleanolic acid-producing strains varying far less than the BAS strains. The observed reductions in growth may result from (i) amino acid burden caused by expression of the recombinant proteins, (ii) aberrant activities of the transgenic enzymes, (iii) accumulation of triterpenoid product and/or (iv) depletion of native metabolites (e.g. redirection of metabolic flux from sterol to triterpenoid biosynthesis) [3,29]. Most likely, a combination of these factors and others could explain the differences in growth observed between the strains.

The strains used in this study were derived from a BY4741 parent strain transformed with plasmids expressing the triterpenoid biosynthetic gene(s). These therefore represent unoptimised strains and numerous strain engineering approaches could be taken to improve titres. In an early study, overexpression of a truncated version of the native *tHMG1* (a rate-limiting enzyme of the mevalonate pathway required for the production of 2,3-oxidosqualene), and downregulation of the native *ERG7* (lanosterol synthase which uses 2,3-oxidosqualene as substrate), increased β-amyrin titres in yeast expressing *AaBAS* by 1.5-fold (to 6 mg/L), compared with expression of *AaBAS* alone [30]. Higher titres of 50 mg/L β-amyrin were later achieved by expressing *GgBAS* in a strain overexpressing *tHMG1* and with repression of the *ERG7* gene [6]. The strain was cultured in the presence of methyl-β-cyclodextrin, which improved titres, possibly by sequestering β-amyrin in the culture media and preventing toxic build-up within the cells. Meanwhile, another study reported oleanolic acid titres of 71 mg/L [7]. Two copies of *GgBAS* were co-expressed with *CYP716A12* and *AtATR1*, together with overexpression of *tHMG1* and the native genes *ERG9* and *ERG1*, which produce squalene from farnesyl pyrophosphate (FPP, a product of the mevalonate pathway) and convert it to 2,3-oxidosqualene, respectively. In addition, the strains were grown in rich medium (YPD), which may have facilitated growth and the production of triterpenoids. An organelle engineering approach increased titres of β-amyrin and its derivative 28-*O*-glucose-medicagenic acid by 8- and 16-fold compared to an unengineered strain [8]. Knock-out of the *PAH1* gene, encoding phosphatidic acid phosphatase, resulted in proliferation of the ER and a corresponding increase in the intracellular levels of triterpenoid biosynthetic enzymes, boosting strain productivity. The use of fermenter apparatus has also resulted in increased triterpenoid production. For example, oleanolic acid titres of 607 mg/L were achieved in a fermenter, compared with 186 mg/L in shake flasks using the same strain [31].

This study compares BAS and CYP716A enzmye productivities in yeast, reports a range of product specificities between CYP716A enzymes, and identifies that expression of *AsBAS* has minimal impact on yeast growth while producing relatively high titres of β-amyrin. This work should therefore serve as an important resource for the production of industrially relevant triterpenoids in yeast.

## Supporting information

S1 Fig Multiple sequence alignment of GhBAS with functionally characterized BASs. The QW (orange), MWCYCR (blue) and DCTAE (red) motifs are highlighted. The consensus is shown under the alignment as identical residues (*), conserved substitutions (.) and semi-conserved substitutions (:). Protein sequences were aligned using Clustal Omega (www.ebi.ac.uk/Tools/msa/clustalo/) using default parameters. The BAS protein sequences were retrieved from GenBank: AaBAS, ACA13386.1; AsBAS, CAC84558.1; BvBAS, AFF27505.1; CqBAS1, ANY30852.1; EtBAS, BAE43642.1; GgBAS, BAA89815.1; GhBAS, XP_016749748.1; LjBAS, BAE53429.1; MtBAS, CAD23247.1, PgBAS, BAA33461.1, PtBAS, ABL07607.1; SlBAS, ADU52574.1; TcBAS, XP_017979420.
(DOCX)

S2 Fig Multiple sequence alignment of CYP716A proteins. Protein sequences were aligned using Clustal Omega (www.ebi.ac.uk/Tools/msa/clustalo/) with default parameters. Conserved sequences are highlighted as transmembrane domain (green) predicted by the TMHMM server (http://www.cbs.dtu.dk/services/TMHMM); proline cluster that forms a hinge between the transmembrane and cytoplasmic domains (P450 consensus PPxP, purple); A/G-G-X-D/E-T-T/S motif (proton transfer groove, light blue); ExxR motif (blue); PERF motif (red); FxxGxRxCxG motif (heme binding loop, orange). The sequences contained an additional ‘GS’ at the C-terminus, but were otherwise identical to the GenBank sequences. The protein sequences were retrieved from GenBank: CYP716A1, NP_198460.1; CYP716A12, CBN88269.1; CYP716A15, BAJ84106; CYP716A44, AK329870.1; CYP716A48, BAP59949.1; CYP716A52v2, AFO63032; CYP716A75, AHF22088.1; CYP716A79, ANY30854.1; CYP716A80, ALR73782.1; CYP716A83, AOG74832.1; CYP716A110, AOG74847.1; CYP716A140, AOG74836.1; CYP716A147, XP_007023618; CYP716A179, BAW34647.1; CYP716A244, APZ88353.1; CYP716AL1, AEX07773.
(DOCX)

S3 Fig BvBAS is a multifunctional OSC producing multiple triterpenes. (A) Total ion chromatogram (TIC) of strains expressing AaBAS, BvBAS and the MD-N1 control strain (expressing no BAS). While both AaBAS and BvBAS have a peak corresponding to β-amyrin, the BvBAS strain has a second unique peak (*) that is potentially a combination of two peaks and which is not present in either the AaBAS or control strains. (B) Mass spectrum at retention time marked by asterix (*) (top). Comparison with a mass spectrum of an authentic lupeol standard (bottom) suggests one of the compounds contained within the peak corresponds to lupeol.
(DOCX)

S1 Table List of enzymes. (DOCX)
