# Supplementary material for: A systematic comparison of triterpenoid biosynthetic enzymes for the production of oleanolic acid in Saccharomyces cerevisiae
Source: PLoS One. 2020 May 1;15(5):e0231980. doi: 10.1371/journal.pone.0231980 (PMC7194398; doi:10.1371/journal.pone.0231980)
Supplement: S1 Fig — The QW (orange), MWCYCR (blue) and DCTAE (red) motifs are highlighted. The consensus is shown under the alignment as identical residues (*), conserved substitutions (.) and semi-conserved substitutions (:). Protein sequences were aligned using Clustal Omega (www.ebi.ac.uk/Tools/msa/clustalo/) using default parameters. The BAS protein sequences were retrieved from GenBank: AaBAS, ACA13386.1; AsBAS, CAC84558.1; BvBAS, AFF27505.1; CqBAS1, ANY30852.1; EtBAS, BAE43642.1; GgBAS, BAA89815.1; GhBAS, XP_016749748.1; LjBAS, BAE53429.1; MtBAS, CAD23247.1, PgBAS, BAA33461.1, PtBAS, ABL07607.1; SlBAS, ADU52574.1; TcBAS, XP_017979420. (DOCX) [file pone.0231980.s001.docx]

**Supplementary Figure S1**

AsBAS MWRLTIGEGG--GPWLKSNNGFLGRQVWEYDADA-GTPEERAEVERVRAEFTKNRFQRKE 57

BvBAS MWRLKLGEGNGDDPYLFSSNNFVGRQTWEFDPKA-GTLEERAAVEEARRSFLVNRSRVKA 59

CqBAS1 MWRLKVGEGA-NDPYLYSTNNFVGRQTWEFDPNY-GTPEEREEVEEARRNFYNNRFKVKP 58

AaBAS MWRLKIAEGR-NDPYLYSTNNFVGRQIWEFDPNY-GTPEERAEVEQARVDFWNHRHEVKP 58

PtBAS MWRLKVGEGK-NDPYLFSTNDYTGRQTWEFDPDA-GTPEERAEVEAARQAFYDNRFQFKN 58

MtBAS MWKLKIGEGK-NEPYLFSTNNFVGRQTWEYDPEA-GSEEERAQVEEARKNFYDNRFKVKP 58

GgBAS MWRLKIAEGG-KDPYIYSTNNFVGRQTWEYDPDG-GTPEERAQVDAARLHFYNNRFQVKP 58

LjBAS MWKLKVADGG-KDPYIFSTNNFVGRQTWEYDPDA-GTPEERAQVEEARQDFYNNRYKVKP 58

PgBAS MWKLKIAEGNKNDPYLYSTNNFVGRQTWEFDPDYVASPGELEEVEQVRRQFWDNRYQVKP 60

SlBAS MWKLKIAEGQ-NGPYLYSTNNYVGRQTWEFDPNG-GTIEERAKIEEARQQFWNNRYKVKP 58

EtBAS MWKLKIAEGG-NDEYLYSTNNYVGRQTWVFDPQP-PTPQELAQVQQARLNFYNNRYHVKP 58

GhBAS MWKLKVAEGV-DGPYLYSTNNYVGRQTWEFDPDS-GTPEERAQVEEARKNFYKNRHHVKP 58

TcBAS MWKLKIAEGV-DGPYLYSTNNYVGRQTWEFDPDA-GTPEERAEVEEARQNFYKDRYQVKP 58

**:*.:.:* :: *.*.: *** * :* . : * :: .* * .* . *

AsBAS SQDLLLRLQYAKDNPLPANIPTEAKLEKSTEVTHETIYESLMRALHQYSSLQADDGHWPG 117

BvBAS CSDLLWRMQFLKEAKFEQVIPP-VKIEDAKDITYENATDSLRRGVSFFSALQASDGHWPG 118

CqBAS1 CGDLIWRLQFLREKNFKQTIPQ-VKVEEGEEITYETATTTLKRAVNVFTALQSDQGHWPA 117

AaBAS SSDVLWRMQFLREKGFEQTIPQ-VKIEDGEEISYEKATTTLRRSVNFFAALQADDGHWPA 117

PtBAS CGDLLWRFQFLRDKNFKQTIPK-VKVEDGQQITYEMATDTVRRAAHHLGGLQSSHGHWPA 117

MtBAS CGDLLWRFQVLRENNFMQTIDG-VKIEDGEEITYEKATTTLRRGTHHLAALQTSDGHWPA 117

GgBAS CGDLLWRFQILRENNFKQTIAS-VKIGDGEEITYEKATTAVRRAAHHLSALQTSDGHWPA 117

LjBAS CGDLLWRFQVLRENNFKQTIPS-VKIEDGEEITYEKATTTLKRAAHHLAALQTSDGHWPA 117

PgBAS SGDLLWRMQFLREKNFRQTIPQ-VKVGDDEAVTYEAATTTLRRAVHFFSALQASDGHWPA 119

SlBAS SSDLLWRIQFLGEKNFKQKIPA-VKVEEGEEISHEVATIALHRAVNFFSALQATDGHWPA 117

EtBAS SSDLLWRFQFLREKNFKQTIPQ-AKINEGEDITYEKATTALRRAVHFFSALQASDGHWPA 117

GhBAS SADLLWRMQFLKEKNFKQSIPA-VKIDDGEQITYEKATTTLRRAVHFFSALQASDGHWPA 117

TcBAS SGDLLWRMQFLREKNFKQTIPA-VKIEEGEQISYEKATAALRRAVHFFSALQASDGHWPA 117

. *:: *:* : : * .*: . :::* :: *. .**: .****.

AsBAS DYSGILFIMPIIIFSLYVTRSLDTFLSPEHRHEICRYIYNQQNEDGGWGKMVLGPSTMFG 177

BvBAS EIAGPLFFLPPLVFCLYITGHLEEIFDEEHRKEMLRHVYCHQNEDGGWGLHVESKSIMFC 178

CqBAS1 EIAGPQFFLPPLVFCLYITGDLNSVFGPEHRREILRSIYYHQNEDGGWGLHIEGHSTMFC 177

AaBAS ENAGPLYFMQPLVICLYITGHLNTVFPAEYRKEILRYIYCHQNEDGGWGFHIEGHSTMFC 177

PtBAS QIAGPLFFMPPLVFCLYITGHLNTVFPEEHRKEILRYIYYHQNEDGGWGLHIEGHSTMFC 177

MtBAS QIAGPLFFMPPLVFCVYITGHLDSVFPREHRKEILRYIYCHQNEDGGWGLHIEGHSTMFC 177

GgBAS QIAGPLFFLPPLVFCMYITGHLDSVFPEEYRKEILRYIYYHQNEDGGWGLHIEGHSTMFC 177

LjBAS QIAGPLFFQPPLVFCMYITGHLNSVFPEEYRKEILRYIYVHQNEDGGWGLHIEGHSTMFC 177

PgBAS ENSGPLFFLPPLVMCVYITGHLDTVFPAEHRKEILRYIYCHQNEDGGWGLHIEGHSTMFC 179

SlBAS ENAGPLFFLPPLVMCMYITGHLNTVFPAEHRKEILRYIYCHQNEDGGWGLHIEGHSTMFC 177

EtBAS ENAGPLFFLPPLVMCLYITGHLDTVFPAPHRLEILRYIYCHQNEDGGWGLHIEGHSTMFC 177

GhBAS ENAGPLFFLPPLVFSMYITGHLNTVFPEEHRREILRYIYYHQNEDGGWGLHIEGHSTMFC 177

TcBAS ENAGPLFFLPPLVFSTYITGHLNTVFPEEHRREILRYIYYHQNEDGGWGLHIEGHSTMFC 177

: :* :: :::. *:* *: .: :* *: * :* :******** : . * **

AsBAS SCMNYATLMILGEKRNGDHKDALEKGRSWILSHGTATAIPQWGKIWLSIIGVYEWSGNNP 237

BvBAS TVLNYICLRMLGEGPNGGRDNACKRARQWILDRGGVTYIPSWGKIWLSILGIYDWSGTNP 238

CqBAS1 TALNYICLRMLGIGPDEGDDNACPRARKWILDHGSVTHMPSWGKTWLSILGLFDWSGSNP 237

AaBAS TTLSYICMRLLGEGRDGGLDGACTKARKWILDHGSVTTIPSWGKTWLSILGVCEWAGTNP 237

PtBAS TALSYICMRMLGEGPEGGLNNACVRARKWILDHGGVTHIPSWGKTWLSVLGIFDWSGSNP 237

MtBAS TALNYICMRILGEGPDGGQDNACARARNWIRAHGGVTYIPSWGKTWLSILGLFDWLGSNP 237

GgBAS TALNYICMRILGEGPDGGQDNACARARKWIHDHGGVTHIPSWGKTWLSILGVFDWCGSNP 237

LjBAS TALNYICMRMLGEGPDGGQDNACARARKWILDHGGVTHIPSWGKTWLSILGIFDWKGSNP 237

PgBAS TTLSYICMRILGEGPDGGVNNACARGRKWILDHGSVTAIPSWGKTWLSILGVYEWIGSNP 239

SlBAS TALSYICMRILGEGPDGGVNNACARARKWILDHGSVTAIPSWGKTWLSILGVFEWIGTNP 237

EtBAS TVLSYICMRLLGEGPNGGQDNACSRARKWIIDHGGATYIPSWGKTWLSILGVYEWSGSNP 237

GhBAS TALSYICMRILGEGPDGGLDNACARARKWILDHGSVTHMPSWGKTWLSILGVFDWSGCNP 237

TcBAS TALSYICMRILGVEPDGGQDNACARARKWILDHGSVTHMPSWGKTWLSILGVFDWSGSNP 237

: :.* : :** : . ..* :.*.** :* .* :*.*** ***::*: :* * **

AsBAS IIPELWLVPHFLPIHPGRFWCFTRLIYMSMAYLYGKKFVGPISPTILALRQDLYSIPYCN 297

BvBAS MPPEIWLLPSFVPIHLAKTLCYCRMVYMPMSYLYGKRFVGPITPLILQLREELHLQPYEA 298

CqBAS1 MPPEFWLLPSFLPMYPAKMWCYCRMVYMPMSYLYGKRFIGPITPLIKELREELYNEPFEQ 297

AaBAS MPPEFWILPSFLPMYPAKMWCYCRLVYMPMSYLYGKRFVGPITPLILQLRDELYAQPYDE 297

PtBAS MPPEFWILPSFLPMHPAKMWCYCRMVYMPMSYLYGKRFVGPITPLIKQLREELFTQPFEE 297

MtBAS MPPEFWILPSFLPMHPAKMWCYCRLVYMPMSYLYGKRFVGPITPLILQLREELHTQPYEK 297

GgBAS MPPEFWILPSFLPMHPAKMWCYCRLVYMPMSYLYGKRFVGPITPLILQLREELFTEPYEK 297

LjBAS MPPEFWILPSFLPMHPAKMWCYCRLVYMPMSYLYGKRFVGPITPLILQLREELFTQPYEK 297

PgBAS MPPEFWILPSFLPMHPAKMWCYCRMVYMPMSYLYGKRFVGPITPLILQLREELYGQPYNE 299

SlBAS MPPEFWILPSFLPVHPAKMWCYCRMVYMPMSYLYGKRFVGPITPLILQLREELYDRPYDE 297

EtBAS MPPEFWILPTFLPMHPAKMWCYCRMVYMPMSYLYGKRFVGPITPLILQLRQELHTQPYHH 297

GhBAS MPPEFWLLPSFLPMHPAKMWCYCRMVYMPMSYLYGKRFVGPITPLIEQLREELYLQPYNE 297

TcBAS MPPEFWILPSFLPMHPAKMWCYCRMVYMPMSYLYGKRFVGPITPLIEQLREELYLQPYNE 297

: **:*::* *:*:: .: *: *::** *:*****:*:***:* * **::*. *:

AsBAS INWDKARDYCAKEDLHYPRSRAQDLISGCLTKIVEPILNWWPANKL-RDRALTNLMEHIH 356

BvBAS INWNKTRRLYAKEDMYFPHPLVQDLIWDTLHIFVEPLLTHWPLNKLVREKALRLAMKHIH 358

CqBAS1 ISWKEMRHLCAPEDLYYPHPLIQDLMWDALYIFTEPLLTRWPFNKLIRKKALEVTMEHIH 357

AaBAS IKWRSIRHLCAKEDLYYPHPLLQDLMWDSLYVFTEPVLNHWPFNKL-REKALQTTMKHIH 356

PtBAS INWKKARHQCASEDIYYPHPWVQDLIWDTLYICSEPLLTRWPFNKLIREKALQVTMKHIH 357

MtBAS INWTKSRHLCAKEDIYYPHPLIQDLIWDSLYIFTEPLLTRWPFNKLVRKRALEVTMKHIH 357

GgBAS VNWKKARHQCAKEDLYYPHPLLQDLIWDSLYLFTEPLLTRWPFNKLVREKALQVTMKHIH 357

LjBAS VNWKKARHQCAKEDIYYPHPLIQDLMWDSLYLFTEPLLTRWPFNKLVREKALEVTMKHIH 357

PgBAS INWRKTRRVCAKEDIYYPHPLIQDLLWDSLYVLTEPLLTRWPFNKL-REKALQTTMKHIH 358

SlBAS INWKKVRHVCAKEDLYYPHPLVQDLMWDSLYICTEPLLTRWPFNKL-RNKALEVTMKHIH 356

EtBAS INWTKTRHLCAHEDVYYPHPLIQDLMWDSLYIFTEPLLTRWPFNKIIRKKALEVTMKHIH 357

GhBAS INWKKIRHLCAPEDIYYPHPLIQDLMWDSLYICTEPLLTRWPFNKLIRERTLQVTMKHIH 357

TcBAS INWRKVRHLCAPEDIYYPHPLIQDLMWDSLYICTEPLLTRWPLNKLVREKALQVTIKHIH 357

:.* . * * **:::*: ***: . * **:*. ** **: *.::* ::***

AsBAS YDDESTKYVGICPINKALNMICCWVENPNSPEFQQHLPRFHDYLWMAEDGMKAQVYDGCH 416

BvBAS YEDENSHYITIGCVEKVLCMLACWIDDPNGDYFKKHLARIPDYMWVAEDGMKMQSF-GSQ 417

CqBAS1 YEDENSRYITIGCVEKVLCMLACWVEDPKGDHYKKHLARVQDYIWIAEDGLKMQSF-GSQ 416

AaBAS YEDENSRYITIGSVEKALCMLACWVEDPNGVCFKKHIARIPDYLWVAEDGMKMQSF-GSQ 415

PtBAS YEDENSRYITIGCVEKVLCMLACWVEDPNGDAYKKHLARVPDYLWLSEDGMCVQSF-GSQ 416

MtBAS YEDENSRYLTIGCVEKVLCMLACWVEDPNGDAYKKHLARVQDYLWMSEDGMTMQSF-GSQ 416

GgBAS YEDETSRYITIGCVEKVLCMLACWVEDPNGDAFKKHLARVPDYLWVSEDGMTMQSF-GSQ 416

LjBAS YEDENSRYITIGCVEKVLCMLACWVEDPNGDAFKKHLARIPDYLWVSEDGMCMQSF-GSQ 416

PgBAS YEDENSRYITIGCVEKVLCMLVCWVEDPNGDYFRKHLARIPDYIWVAEDGMKMQSF-GSQ 417

SlBAS YEDENSRYITIGCVEKVLCMLACWVEDPNGDYFKKHLARIPDYLWVAEDGMKMQSF-GSQ 415

EtBAS YEDENSRYITIGCVEKVLCMLACWAEDPNGVPFKKHLARIPDYMWVAEDGMKMQSF-GSQ 416

GhBAS YEDENSRYITIGCVEKVLCMLACWAEEPNSDYFKKHLARIPDYLWVAEDGMKMQSF-GSQ 416

TcBAS YEDENSRYITIGCVEKVLCMLACWAEEPNSDYFKKHLARIPDYLWVAEDGMKMQSF-GSQ 416

*:**.::*: * ::*.* *: ** ::*:. :::*: *. **:*::***: * : *.:

AsBAS SWELAFIIHAYCSTDLT-SEFIPTLKKAHEFMKNSQVLFNHP-NHESYYRHRSKGSWTLS 474

BvBAS QWDTGFAVQAIIASDLS-SETGDVLKRGHDYIKKSQIRENPSGDFKSMYRHISKGAWTLS 476

CqBAS1 EWDCGFSVQALLASNLSLDEIGPALKKGHFFIKESQVKDNPSGDFKAMHRHISKGSWTFS 476

AaBAS EWDAGFAIQALMATDLT-DEIGSTLMKGHEFIKASQVKDNPSGDFKSMHRHISKGSWTFS 474

PtBAS EWDAGFAVQALLAANLV-DEIAPVLAKGHDFIKKSQVKDNPSGDFKSMHRHISKGSWTFS 475

MtBAS EWDAGFAVQALLAANLN-DEIEPALAKGHDFIKKSQVTENPSGDFKSMHRHISKGSWTFS 475

GgBAS EWDAGFAVQALLATNLV-EEIAPTLAKGHDFIKKSQVRDNPSGDFKSMYRHISKGSWTFS 475

LjBAS EWDAGFAVQALLATNLV-DELGPTLAKGHDFIKKSQVRDNPSGDFKNMHRHISKGSWTFS 475

PgBAS EWDTGFSIQALLDSDLT-HEIGPTLMKGHDFIKKSQVKDNPSGDFKSMYRHISKGSWTFS 476

SlBAS EWDTGFAIQALLASEMN-DEIADTLRKGHDFIKQSQVTNNPSGDFKGMYRHISKGSWTFS 474

EtBAS QWDTGFAIQALLASNLT-EEIGQVLKKGHDFIKKSQVKENPSGDFKSMHRHISKGSWTFS 475

GhBAS QWDTGFAIQALLASNLT-DEIGPVLKRGHDFIKKSQVKDNPSGDFKQMFRHISKGSWTFS 475

TcBAS EWDTGFAIQALLASNLT-DEIGPVLKRGHDFIKKSQVKDNPSGDFKKMYRHISKGSWTFS 475

.*: .* ::* ::: * .* :.* ::* **: * :.: .** ***:**:*

AsBAS SVDNGWSVSDCTAEAVKALLLLSKISADLVGDPIKQDRLYDAIDCILSFMNTDGTFSTYE 534

BvBAS DRDHGWQVSDCTAEALKCCLLLSMMPAEVVGHKMDPEQLYDSVNLLLSLQSANGGVTAWE 536

CqBAS1 DQDHGWQVSDCTAEGLKCCLILSTMPLEIVGEKMDPERLYDSVNVLLSLQSKNGGLAAWE 536

AaBAS DQDHGWQVSDCTAEALKCCLLFATMPPEIVGEKMKPEQLNDAVNVILSLQSKNGGLAAWE 534

PtBAS DQDHGWQVSDCTAEGLKVCLQMSLLPPEIVGEKMEPERLFDSVNVLFSLQSKKGGLAAWE 535

MtBAS DQDHGWQVSDCTAEGLKCCLLLSMLPPEIVGEKMEPERLYDSVNVLLSLQSKKGGLAAWE 535

GgBAS DQDHGWQVSDCTAEGLKCCLLLSMLPPEIVGEKMEPERLYDSVNVLLSLQSKKGGLSAWE 535

LjBAS DQDHGWQVSDCTAEGLKCCLLLSMLPPDIVGEKMEPECLFDSVNLLLSLQSKKGGLAAWE 535

PgBAS DQDHGWQVSDCTAEGLKCCLIFSTMPEEIVGKKIKPERLYDSVNVLLSLQRKNGGLSAWE 536

SlBAS DQDHGWQVSDCTAEALKCCLLLSTMPRELVGQAMEPGRLYDSVNVVLSLQSKNGGLAAWE 534

EtBAS DQDHGWQVSDCTAEGLKCCLLFSMMPPEIVGEKMDAQHLYNAVNILISLQSKNGGLAAWE 535

GhBAS DQDHGWQVSDCTAEGLKCCLLMSMLPPEIVGEKMEPQQLYDAVNVILSLQSQNGGLAAWE 535

TcBAS DQDHGWQVSDCTAEGLKCCLLMSMLPPEIVGEKMEPQQLYNAVNVLLSLQSKNGGLAAWE 535

. *:**.*******.:* * :: : ::**. :. * :::: ::*: .* .:::*

AsBAS CKRTFAWLEVLNPSESFRNIVVDYPSVECTSSVVDALILFKETNPRYRRAEIDKCIEEAV 594

BvBAS PVRAYAWTELLNPTEFLANLVAEREYVECTSSVVQALVLFQQLYPDHKTKKISRAIEKAV 596

CqBAS1 PAGAQEWLEVLNPTEFFEGIVIEYEYVECTASAIQALVMFKKLYPGHRKKEIDNFVVNAV 596

AaBAS PAGSSEWLEILNPTEFFADIVIEHEYVECTSSAIQALVMFKKKYPGHRKKEIENFLLGSS 594

PtBAS PAGAQEWLELLNPTEFFADIVVEHEYVECTGSAIQALVLFKKLYPGHRKKEIDNFIINAV 595

MtBAS PAGAQEWLELLNPTEFFADIVVEHEYVECTGSAIQALVLFKKLYPGHRKKEIENFISEAV 595

GgBAS PAGAQEWLELLNPTEFFADIVVEHEYVECTGSAIQALVLFKKLYPGHRKKEIENFIANAV 595

LjBAS PAGAQEWLELLNPTEFFADIVVEHEYVECTGSAIGALVLFKKLYPGHRKKEIENFISEAV 595

PgBAS PAGAQEWLELLNPTEFFADIVIEHEYVECTSSAIQALVLFKKLYPGHRKKEIDNFITNAV 596

SlBAS PAGASEYLELLNPTEFFADIVIEHEYVECTASSIQALVLFKKLYPGHRTKEINIFIDNAV 594

EtBAS PAGAQQWLEMLNPTEFFADIVIEHEYVECTASAIHALIMFKKLYPGHRKKEIENFITNAV 595

GhBAS PAGAQEWLEMLNPTEFFADIVIEHEYIECTASSIHALVMFKKLYPGHRKKEIDNFITNAV 595

TcBAS PAGAQDWLEMLNPTEFFADIVVEHEYVECTASAIHALFLFKKLYPGHRKKEIDNFITNAV 595

: : *:***:* : .:* : :***.* : **.:*:: * :: :*. : :

AsBAS VFIENSQNKDGSWYGSWGICFAYGCMFAVRALVATGKTYDNCASIRKSCKFVLSKQQTTG 654

BvBAS QFLENEQKPDGSWYGNWGVCFIYATWFALGGLAAAGKTYKTSQAMRKGVEFLLTTQKDDG 656

CqBAS1 RYLENTQFPNGGWYGNWGICFIYGTWFALGGLAAGGKTYYNCAAVRKGVEFLLTTQKEDG 656

AaBAS GYLEKIQMEDGSWYGNWGVCFTYGTWFALGGLSAVGKTYDNCPAIRKAVKFLLETQLEDG 654

PtBAS RFLEDTQTADGSWYGNWGVCFTYGSWFALGGLAAAGKTFSNCAAIRKAVHFLLTTQKEDG 655

MtBAS RFIEDIQTADGSWYGNWGVCFTYGSWFALGGLAAAGKTYTNCAAIRKAVKFLLTTQREDG 655

GgBAS RFLEDTQTADGSWYGNWGVCFTYGSWFALGGLAAAGKTFANCAAIRKAVKFLLTTQREDG 655

LjBAS RFLEDTQTADGSWYGNWGVCFTYGSWFALGGLAAAGKTYANCAAIRKAVKFLLTTQRGDG 655

PgBAS RYLEDTQMPDGSWYGNWGVCFTYGSWFALGGLAAAGKTYYNCAAVRKAVEFLLKSQMDDG 656

SlBAS KYLEDVQMPDGSWYGNWGVCFTYGSWFALGGLVAAGKSYNNSAAVRKGVEFLLRTQRSDG 654

EtBAS KYLEDVQTADGGWYGNWGVCFTYGTWFAVGGLAAAGKNYNNCAAMRKAVDFLLRTQKQDG 655

GhBAS HYLEDIQMPDGSWYGNWGVCFTYGTWFALGGLAAAGKTYTNCEAMRRGVQFLLTTQRENG 655

TcBAS RYLENIQMPDGSWYGNWGVCFTYGSWFALGGLAAAGKTYTNCLAVRKGVEFLLRTQRENG 655

::*. * :*.***.**:** *. **: .* * **.: .. ::*:. .*:* .* *

AsBAS GWGEDYLSSDNGEYI--DSGRPNAVTTSWAMLALIYAGQVERDPVPLYNAARQLMNMQLE 712

BvBAS GWGESYLSCPEQRYIPLEGNRSNLVQTAWAIMGLIHAGQAERDPIPLHRAAKLIINSQME 716

CqBAS1 GWGESYISCPKKEFVPIEG-KSNLVQTGWALMGLLHAGQAERDPTPLHRAAKLLINSQLE 715

AaBAS GWGESYKSCPEKKYIPLEGGRSNLVHTAWAMMGLIHSRQAERDATPLHRAAKLLINSQLE 714

PtBAS GWGESYLSSPKKIYVPLEISRSNVVQTAWAMMGLIHAGQADRDPTPLHRAAKLLINAQLE 715

MtBAS GWGESYLSSPKKIYVPLEGSRSNVVHTAWALMGLIHAGQAERDPTPLHRAAKLLINSQLE 715

GgBAS GWGESYLSSPKKIYVPLEGSRSNVVHTAWALMGLIHAGQAERDPAPLHRAAKLIINSQLE 715

LjBAS GWGESYLSSPKKIYVPFEGNRSNVVHTAWALMGLIHSGQAERDPTPLHRAAKLLINSQLE 715

PgBAS GWGESYLSCPKKVYVPLEGNRSNLVHTGWALMGLIHSEQAERDPTPLHRAAKLLINSQME 716

SlBAS GWGESYRSCPDKVYRELETNDSNLVQTAWALMGLIHSGQADRDPKPLHRAAKLLINSQME 714

EtBAS GWGESYLSCPHKKYVPLEDNRSNLVHTSWALMGLISAGQMDRDPTPLHRAAKLLINSQLE 715

GhBAS GWGESYKSCPEKRYVPLEDGRSNLVHTAWAMMGLIHAGQAERDPRPLHRAAKLIINSQLE 715

TcBAS GWGESYKSCPDKRYVPLEEGRSNLVHTAWAMMGLIHAGQAERDPTPLHRAAKLIINSQLE 715

****.* *. . : : * * *.**::.*: : * :** **:.**: ::* *:*

AsBAS TGDFPQQEHMGCFNSSLNFNYANYRNLYPIMALGELRRRLLAIKS----- 757

BvBAS NGDFPQQEIVGVFMRNCLLHYATFRNTFPLWALAEYRKAAFVTHKH---- 762

CqBAS1 NGDFPQQEITGVFMKNCMLHYPMYRSIYPMWALAEYRKRVSLPSINSA-- 763

AaBAS TGDFPQQEIAGVFMKNCMLHYALYRNIYPMWALADYRKQVLPQLKGT--- 761

PtBAS NGDWPQQEVTGVFMKNCMLHYPMYRNIYPMWALAEYKRRVPLPSNAS--- 762

MtBAS EGDWPQQEITGVFMKNCMLHYPMYRDIYPLWALAEYRRRVPLPSTAV--- 762

GgBAS EGDWPQQEITGVFMKNCMLHYPMYRDIYPMWALAEYRRRVPLPSTPVCLT 765

LjBAS EGDWPQQEITGVFMKNCMLHYPMYRDIYPMWALAEYRRRVPLPSTAV--- 762

PgBAS DGDFPQQEISGVFMKNCMLHYAAYRNIYPLWALAEYRRRVPLPSLGT--- 763

SlBAS DGDFPQQEITGVFMKNCMLHYAAYRNIYPLWGLAEYRKNVLLPLENN--- 761

EtBAS DGDFPQQEITGVFMKNCMLHYAAYRNIYPLWALAEYRNRVPLPSTTL--- 762

GhBAS DGDFPQQEITGVFMKNCMLHYAAYRNIYPLWALAEYRKRVPLP------- 758

TcBAS DGDFPQQEITGVFMKNCMLHYAAYRNIYPLWALAEYRKRVPLA------- 758

**:**** * * . ::* :*. :*: .*.: :.

**Fig. S1.** **Multiple sequence alignment of GhBAS with functionally characterized BAS proteins.** The QW (orange), MWCYCR (blue) and DCTAE (red) motifs are highlighted. The consensus is shown under the alignment as identical residues (*), conserved substitutions (.) and semi-conserved substitutions (:). Protein sequences were aligned using Clustal Omega (www.ebi.ac.uk/Tools/msa/clustalo/) using default parameters. The BAS protein sequences were retrieved from GenBank: AaBAS, ACA13386.1; AsBAS, CAC84558.1; BvBAS, AFF27505.1; CqBAS1, ANY30852.1; EtBAS, BAE43642.1; GgBAS, BAA89815.1; GhBAS, XP_016749748.1; LjBAS, BAE53429.1; MtBAS, CAD23247.1, PgBAS, BAA33461.1, PtBAS, ABL07607.1; SlBAS, ADU52574.1; TcBAS, XP_017979420.
