# Supplementary material for: A systematic comparison of triterpenoid biosynthetic enzymes for the production of oleanolic acid in Saccharomyces cerevisiae
Source: PLoS One. 2020 May 1;15(5):e0231980. doi: 10.1371/journal.pone.0231980 (PMC7194398; doi:10.1371/journal.pone.0231980)
Supplement: S2 Fig — Protein sequences were aligned using Clustal Omega (www.ebi.ac.uk/Tools/msa/clustalo/) with default parameters. Conserved sequences are highlighted as transmembrane domain (green) predicted by the TMHMM server (http://www.cbs.dtu.dk/services/TMHMM); proline cluster that forms a hinge between the transmembrane and cytoplasmic domains (P450 consensus PPxP, purple); A/G-G-X-D/E-T-T/S motif (proton transfer groove, light blue); ExxR motif (blue); PERF motif (red); FxxGxRxCxG motif (heme binding loop, orange). The sequences contained an additional ‘GS’ at the C-terminus, but were otherwise identical to the GenBank sequences. The protein sequences were retrieved from GenBank: CYP716A1, NP_198460.1; CYP716A12, CBN88269.1; CYP716A15, BAJ84106; CYP716A44, AK329870.1; CYP716A48, BAP59949.1; CYP716A52v2, AFO63032; CYP716A75, AHF22088.1; CYP716A79, ANY30854.1; CYP716A80, ALR73782.1; CYP716A83, AOG74832.1; CYP716A110, AOG74847.1; CYP716A140, AOG74836.1; CYP716A147, XP_007023618; CYP716A179, BAW34647.1; CYP716A244, APZ88353.1; CYP716AL1, AEX07773. (DOCX) [file pone.0231980.s002.docx]

**Supplementary Figure S2**

CYP716A80 ----MYLT-ILFLFVSSILLSLMFLLRKHLSH------FSYQNLPPGKTGFPLIGESLSF 49

CYP716A1 ----MYMAIMIILFLSSILLSLLLLLRKHLSH------FSYPNLPPGNTGLPLIGESFSF 50

CYP716A110 -MEQISLSWLITLVALFLFLPLILLSTRNK-------ANTSNNFPPGKTGWPIIGESLEF 52

CYP716A79 -MELFFLCSIA--LVFFLSLPFLYLFYRHNS-------TRGYKLPPGSMGWPVVGESLEF 50

CYP716A12 MEPNFYLSLLLLFVSF-ISLSLFFIFYK-Q--------KSPLNLPPGKMGYPIIGESLEF 50

CYP716A179 -MEHFYMSLLLLFVTL-VSLSLFFLIFYYNKH----NMNNNNNLPPGKMGYPVIGESLEF 54

CYP716A147 -MDPIIL-LFC--TIFFAAISLGVLLYSKNP------NASHPNLPPGRMGLPLIGESLEY 50

CYP716A75 -MELLFVSLLSLFLLILLPLSLLFLFPSSFSTTTTEANKNSANLPPGLTGWPVVGESFQF 59

CYP716A140 -MELLYVSLLTLLVLL-FPLSLHFLFKKTKS-----GLGEVGRLPPGKTGWPIVGESIEF 53

CYP716A15 -MEVFFLSLLLIFVLS-VSIGLHLLFYKHRS-----HFTG-PNLPPGKIGWPMVGESLEF 52

CYP716A83 -MELFFVPLFSSLVLF-VFFCFLLLFYKNNK-----WRSSGAPLPPGQTGWPFIGESYEF 53

CYP716A52v2 -MELFYVPLLSLFVLF-ISLSFHFLFYKSKP-----SSSGGFPLPPGKTGWPIIGESYEF 53

CYP716A244 -MQLFYVPLLSLFVLL-VSLSFYFLFYKSKS-----GSPGALPLPPGKTGWPVIGESFEF 53

CYP716A44 -MELLYVCLVCVFVFL-VSLLL---LYKKK-------SGE--GLPPGKTGWPVFGESLEF 46

CYP716AL1 -MEIFYVTLLSLFVLL-VSLSFHFLFYKNKS-----TLPG--PLPPGRTGWPMVGESLQF 51

CYP716A48 -MEFFYVSLLCLFVFL-ISLSLHFLFYKNKS-----SFSG--QIPPGKTGWPVIGESLEF 51

: : . : : :*** * *..*** .:

CYP716A80 LSEGSQGHPEKFITDRVRRFISSSSGVFKTHLFGSPTAVMTGASGNKFLFTNENKLVVSW 109

CYP716A1 LSAGRQGHPEKFITDRVRRFSSSSSCVFKTHLFGSPTAVVTGASGNKFLFTNENKLVVSW 110

CYP716A110 LNTGRRGVPEKFIHDRMQKFS---SHIFRTSLLGEPAAVLCGPTGNKFLYSNENKLVQAW 109

CYP716A79 FSTGWKGYPEKFIFDRLKKYKP--SQVFKTSIFGEQVAILCGATGNKFLYSNENKLVQAW 108

CYP716A12 LSTGWKGHPEKFIFDRMRKYS---SELFKTSIVGESTVVCCGAASNKFLFSNENKLVTAW 107

CYP716A179 LSTGWKGHPEKFIFDRMVRYS---SELIKTSILGVPTVIFCGPACNKFLFSNENKLVTGW 111

CYP716A147 LLTGRKGYPEKFLNDRMAKYS---SQVFKTSILGESMAVVCGAAGNKFLFSNENKLVTAW 107

CYP716A75 LAAGWRGNPEKFIFDRIAKYS---SYVYKTNLMLERTAVFCGAPAHKFLFSNENKLVQSW 116

CYP716A140 LSTGWKGHPEKFIFDRMSKYS---PQVFRTSLMLEDAAVFCGSAGNKFLFSNEKQLVTAW 110

CYP716A15 LSTGWKGHPEKFIFDRISKYS---SEVFKTSLLGEPAAVFAGAAGNKFLFSNENKLVHAW 109

CYP716A83 LSTGWKGYPEKFIFDRIAKHS---SNVFKTSILGEHAAVFCGAACNKFLFSNENKLVQAW 110

CYP716A52v2 LSTGWKGYPEKFIFDRMTKYS---SNVFKTSIFGEPAAVFCGAACNKFLFSNENKLVQAW 110

CYP716A244 LSTGWKGHPEKFIFDRLAKYS---SNVFRTSLLGQPAAVFCGAACNKFLFSNENKLVQAW 110

CYP716A44 LSCGWKGHPEKFIFDRVAKYS---SSVFKTHLLGEEAAVFCGASANKFLFSNENKLVQAW 103

CYP716AL1 LSAGWKGHPEKFIFDRMAKYS---SNVFRSHLLGEPAAVFCGAIGNKFLFSNENKLVQAW 108

CYP716A48 LSNGWKGHPEKFIFDRIAKYS---SYVFRTHLFGEPAAVFCGANGNKFLFSNENKLVQAW 108

: * :* ****: **: :. : :: :. .: * :***::**::** .*

CYP716A80 WPDSVKKIFPYTQ-ST-YTEESKKLRILLLQFMKPEALRKYIGVMDEVTQRHFETEWTNK 167

CYP716A1 WPDSVNKIFPSSM-QTSSKEEARKLRMLLSQFMKPEALRRYVGVMDEIAQRHFETEWANQ 169

CYP716A110 WPSSVYKIFPSTG-QSSSSEEAIKMRKMLLSFFKPEALQRYVETMDTIAKQHLKNEWENR 168

CYP716A79 WPKSVDKIFPAAT-QHSSIEEARTMRKLIPLFLKPESLQRYIPIMDTIATRHMESGWEGK 167

CYP716A12 WPDSVNKIFPTTSLDSNLKEESIKMRKLLPQFFKPEALQRYVGVMDVIAQRHFVTHWDNK 167

CYP716A179 WPDSVNKIFPTTS---NSKEESKKMRKLLPQFLKPEALQRYVGIMDTLAQKHFASLWEEK 168

CYP716A147 WPDSVNKIFPSST-QTSSKEESKKMRKMLPNFLKPEALQRYIGMMDNIAQRHFEASWEGK 166

CYP716A75 WPSSVNKIFPSSN-QTSSKEEAMKMRKMLPNFFKPEALQGYIGIMDTIAQRHFAADWDNK 175

CYP716A140 WPASVDKVFPSSD-QR-SKEEAIKMKKLLPTFFKPVALQNYVGVMDQITERHFASDWDNN 168

CYP716A15 WPSSVDKVFPSST-QTSSKEEAKKMRKLLPQFFKPEALQRYIGIMDHIAQRHFADSWDNR 168

CYP716A83 WPDSVNKVFPSST-QTSSKEEAIKMRKMLPNFLKPDALQRYVGTMDSIARRHFESGWDNK 169

CYP716A52v2 WPDSVNKVFPSST-QTSSKEEAIKMRKMLPNFFKPEALQRYIGLMDQIAANHFESGWENK 169

CYP716A244 WPDSVNKVFPSST-QTSSKEEAIKMRKLLPNFLKPEALQRYVGIMDQIAKKHFESGWENK 169

CYP716A44 WPNSVNKVFPSST-QTSSKEEAIKMRKMLPNFFKPEALQRYVGIMDHITQRHFASGWENK 162

CYP716AL1 WPDSVNKVFPSSN-QTSSKEEAIKMRKMLPNFLKPEALQRYIGLMDQIAQKHFSSGWENR 167

CYP716A48 WPASVDKVFPSSN-QTSSKEEAVKMRKMLPTFFKPEALQRYVGIMDHIAQRHFSDGWDNK 167

** ** *:** : **: .:: :: *:** :*: *: ** :: .*: * .

CYP716A80 KQLIVYPLSKKLTFSIACRLFLSMDDPERVSKLEERFNSVVMGIYSIPIDLPGTRFNRSI 227

CYP716A1 DQVIVFPLTKKFTFSIACRSFLSMEDPARVRQLEEQFNTVAVGIFSIPIDLPGTRFNRAI 229

CYP716A110 KEVMVFPLSKGYTFALACKLFMSIDDPIQLARFADPFAILASGVIAIPINLPGTSFNKAI 228

CYP716A79 DKVEVFPLAKRYTFWVACRLFLSIEDPVHVAKFADPFNEIAAGIISIPIDLPGTPFHKGI 227

CYP716A12 NEITVYPLAKRYTFLLACRLFMSVEDENHVAKFSDPFQLIAAGIISLPIDLPGTPFNKAI 227

CYP716A179 THVTVYPLAKRYTFMLACRLFMSVEDENHVAKFREPFHLLASGIISVPIDLPWTPFNRGI 228

CYP716A147 QEITVFPLAKRYTFWVACKVFLSIEDPEHVSKFADPFNALASGIISVPINLPGTPFRRAI 226

CYP716A75 DYIVVFPLCKRYTFWLACKIFMSIEDPKDVDRFLARFNLVAEGLLSIPIDLPGTPFHHSI 235

CYP716A140 NK-VVYPLTKRFTFALACKIFVSIDDPDEVARLVVPFESIATAILSIPLDLPGTGFRRGI 227

CYP716A15 DEVIVFPLAKRFTFWLACRLFMSIEDPAHVAKFEKPFHVLASGLITVPIDLPGTPFHRAI 228

CYP716A83 NEIVVFPLAKTYTFWIACKLFVSVEEPSQVAKLLEPFSAIASGIISVPIDLPGTPFNSAI 229

CYP716A52v2 NEVVVFPLAKSYTFWIACKVFVSVEEPAQVAELLEPFSAIASGIISVPIDLPGTPFNSAI 229

CYP716A244 KEVSVFPLAKNYTFWIACKVFLSVEEPTQVAKLLEPFNAIASGIISVPIDLPGTPFNSAI 229

CYP716A44 EQVVVFPLTKRYTFWLACRLFLSVEDPKHVAKFADPFDVLASGLISIPIDLPGTPFNRAI 222

CYP716AL1 EQVEVFPLAKNYTFWLASRLFVSVEDPIEVAKLLEPFNVLASGLISVPIDLPGTPFNRAI 227

CYP716A48 NEVVVFPLAKRYTFWLACRLFVSVEDPAHVAKFADPFNELASGLISIPIDLPGTPFHRAI 227

*:** * ** :*.: *:*::: : .: * :. .: ::*::** * *. .*

CYP716A80 KASRLIRKEVCAIIGQRREELKAGRASAEQDVLSHMLTSVGE-----TKDEDLANYLIGI 282

CYP716A1 KASRLLRKEVSAIVRQRKEELKAGKALEEHDILSHMLMNIGE-----TKDEDLADKIIGL 284

CYP716A110 KASELIRKDLHKIIKQRKIELAEKRASPMQDVLSHMLSTS-DENGTFMSEMDIADKILGL 287

CYP716A79 KSSEIVRKELRAIIKQRKLDFADGKASPTQDILSHMLLTS-TEDGKFMNEMDIANKILGL 286

CYP716A12 KASNFIRKELIKIIKQRRIDLAEGTASPTQDILSHMLLTS-DENGKSMNELNIADKILGL 286

CYP716A179 KASNFIRKELLKIIRQRKVDLAQGVASPTQDILSHMLLTCDDENGEFMTELNIADKILGL 288

CYP716A147 NASELIRKELMAIIKQRKIDLAENKAAPNQDILSHMLLAT-DENGQYLNELNIADRILGL 285

CYP716A75 KAAEFIREHLVAIIKQRKIDLAEGKASPTQDIMSYMLLTP-DEDGKFMKEYDIADKILGL 294

CYP716A140 NAANFIRKELLAIIKQRKIDLAKGKASPTQDILSHMLLTS-DENGKFMQEYDIADKILGL 286

CYP716A15 KASNFIRKELRAIIKQRKIDLAEGKASQNQDILSHMLLAT-DEDGCHMNEMEIADKILGL 287

CYP716A83 KSSKRIREMLWKIIKQRKIDLAEGKASPTQDILSHMLLTS-DENGKFMSELDIADKILGL 288

CYP716A52v2 KSSKIVRRKLVGIIKQRKIDLGEGKASATQDILSHMLLTS-DESGKFMGEGDIADKILGL 288

CYP716A244 KSSKIIRDKLLGIIKQRKIDLGEGKASPTQDILSHMLLTS-DENGKFMTEGDIADKILGL 288

CYP716A44 KASNFIRKELVRIIKQRKIDLGEGKVSSTQDILSHMLLTC-DENGKFLGDLDIADKILGL 281

CYP716AL1 KASNQVRKMLISIIKQRKIDLAEGKASPTQDILSHMLLTS-DENGKFMHELDIADKILGL 286

CYP716A48 KSSNFIRKELVSIIKQRKIDLAEGKASPTQDILSHMLLTS-DESGKFMHELDIADKILGL 286

:::. :* : *: **: :: . :*::*:** : ::*: ::*:

CYP716A80 LIGGHDTAAIATTFIISYLAEYPHVYQRVLQEQKEILNEKKEKERLKWEDIEKMKYSWNV 342

CYP716A1 LIGGHDTASIVCTFVVNYLAEFPHVYQRVLQEQKEILKEKKEKEGLRWEDIEKMRYSWNV 344

CYP716A110 LIGGHDTASAAITFVMKFLAELPHIYNDVHKEQMEILKSKGSRELLKWEDIQKMKYSWNV 347

CYP716A79 LIGGHDTASASCTFIVKFLAELPHIYEGVYKEQMEIANSKKPGELLNWEDIQKMKYSWNV 346

CYP716A12 LIGGHDTASVACTFLVKYLGELPHIYDKVYQEQMEIAKSKPAGELLNWDDLKKMKYSWNV 346

CYP716A179 LIGGHDTASAACTFIVKYLAELPHIYDRVYQEQMEIANSKSPGELLNWDDINKMRYSWNV 348

CYP716A147 LIGGHDTASAAITFIIKYLAELPDIYNEVYKEQMEIARSKEPGELLNWEDIQKMKYSWNV 345

CYP716A75 LVGGHDTASSACAFIVKYLAELPQVYQGVYKEQMEIAKSKGPGELLNWDDIQKMKYSWNV 354

CYP716A140 LIGGHDTASSACVFIVKYLAELPEIYDGVYKEQMEIAESKAPGELLNWDDLTKMKYSWNV 346

CYP716A15 LIGGHDTASAAITFLIKYMAELPHIYEKVYEEQMEIANSKAPGELLNWDDVQNMRYSWNV 347

CYP716A83 LIGGHDTASSACTFVVKFLAELPEIYDGVYKEQMEIVKSKGPGELLNWDDIQKMKYSWNV 348

CYP716A52v2 LIGGHDTASSACTFVVKFLAELPQIYEGVYQEQMEIVKSKKAGELLKWEDIQKMKYSWNV 348

CYP716A244 LIGGHDTASSACTFVVKFLAELPEIYEGVYKEQMEIAKSKKPGELLSWEDIQKMKYSWNV 348

CYP716A44 LIGGHDTASSACSFIVKYLAELPHIYQRVYTEQMEIAKSKGPGELLRWEDIQKMKYSWNV 341

CYP716AL1 LIGGHDTASSACTFIVKFLGELPEIYEGVYKEQMEIANSKAPGEFLNWEDIQKMKYSWNV 346

CYP716A48 LVGGHDTASSACTFVVKYLAELPEIYEGVYQEQMEIAKSKAPGELLNWDDIQKMKYSWNV 346

*:******: *::.::.* *.:*: * ** ** ..* * * *:*: :*:*****

CYP716A80 ACEVMRLVPPLTGTFREAIDHFTFKGFYIPKGWKLYWSATATHMNPDYFPEPERFEPNRF 402

CYP716A1 ACEVMRIVPPLSGTFREAIDHFSFKGFYIPKGWKLYWSATATHMNPDYFPEPERFEPNRF 404

CYP716A110 ACEVMRLAPPLQGGFREALADFTYNGFSIPKGWKLYWSTNSTHKNSTYFPNPENFDPSRY 407

CYP716A79 ACEVMRLAPPLQGGFREAISDFMYNGFQIPKGWKLYWSANTTHLNPECFPEPQKFDPSRF 406

CYP716A12 ACEVMRLSPPLQGGFREAITDFMFNGFSIPKGWKLYWSANSTHKNAECFPMPEKFDPTRF 406

CYP716A179 ASEVMRVAPPLQGGFREAINDFVFNGFSIPKGWKLYWSANSTHKNPEYFPAPEKFDPTRF 408

CYP716A147 ACEVMRLAPPLQGAFREAITDFTFSGFSIPKGWKLHWNVNSTHKNVECFPEPEKFDPTRF 405

CYP716A75 ACEVLRLAPPLQGAFRDVLKDFMYEGFYIPKGWKVYWSAHSTHKNPEYFPEPYKFDPSRF 414

CYP716A140 ACEVLRLAPPLQGAFREAITDFMYSGFSIPKGWKLYWSANSTHKNPKFFPEPQKFDPSRF 406

CYP716A15 ACEVMRLAPPLQGAFREAITDFVFNGFSIPKGWKLYWSANSTHKSPECFPQPENFDPTRF 407

CYP716A83 ACEVLRLAPPLQGGFREVLTDFSYNGFSIPKGWKIYWTANSTHRNSEVFPEPLKFDPSRF 408

CYP716A52v2 ACEVLRLAPPLQGAFREALSDFTYNGFSIPKGWKLYWSANSTHINSEVFPEPLKFDPSRF 408

CYP716A244 ACEVLRLAPPLQGAFREVLTDFSYNGFSIPKGWKLYWSANSTHRNSEVFPEPLKFDPSRF 408

CYP716A44 ACEVLRLAPPLQGAFREALSDFMFNGFYIPKGWKIYWSANSTHKREEFFPDPEKFDPSRF 401

CYP716AL1 ACEVLRLAPPLQGAFREALNDFMFHGFSIPKGWKIYWSVNSTHRNPECFPDPLKFDPSRF 406

CYP716A48 ACEVLRLAPPLQGAFREAITDFMFNGFSIPKGWKLYWSANSTHRNSEFFPEPLKFDPSRF 406

*.**:*: *** * **:.: .* : ** ******::*.. :** ** * .*:*.*:

CYP716A80 EGSGPKPYTYIPFGGGPRMCPGREFARLEILVIIHNLVNRFKWEKVSPNENKIVVDPLPK 462

CYP716A1 EGSGPKPYTYVPFGGGPRMCPGKEYARLEILIFMHNLVNRFKWEKVFPNENKIVVDPLPI 464

CYP716A110 EGNGPAPYTYVPFGGGPRMCPGKEYARLEILVFMHNVVTKFKWEKILPDE-NIVVNPMPV 466

CYP716A79 DGSGPAPYTFVPFGGGPRMCPGKEYARLEILVFMYHIVKRFKWEKVLPNE-KVIVNPMPI 465

CYP716A12 EGNGPAPYTFVPFGGGPRMCPGKEYARLEILVFMHNLVKRFKWEKVIPDE-KIIVDPFPI 465

CYP716A179 EGNGPAPYTFVPFGGGPRMCPGKEYARLEILVFMHNLVKRFKWEMLIPEE-KIVVDPLPM 467

CYP716A147 EGNGPAPYTFVPFGGGPRMCPGKEYARLEILVFMHNVMKRFNWEKLLPDE-KIIVDPLPM 464

CYP716A75 DGSGPAPYTFVPFGGGPRMCPGKEYARLEILVFMHNLVKRFRWEKLIPDE-KIVVNPMPV 473

CYP716A140 EGAGPAPFTFVPFGGGPRMCPGKEYARVEILVFMHHLVKRFKWEKIIPNE-KIVVNPMPC 465

CYP716A15 EGNGPAPYTFVPFGGGPRMCPGKEYARLEILVFMHNVVKRFKWDKLLPDE-KIIVDPMPM 466

CYP716A83 EGTGPPPFTFVPFGGGPRMCPGKEYARLEILVFIHNLVKRYKWEKIIPDE-KIIVNPMPI 467

CYP716A52v2 DGAGPPPFSFVPFGGGPRMCPGKEYARLEILVFMHHLVKRFKWEKVIPDE-KIVVNPMPI 467

CYP716A244 EGAGPPPYSFVPFGGGPRMCPGKEYARLEILVFMYHVVKRFKWEKVIPDE-KIVVNPMPI 467

CYP716A44 EGSGPAPYTFVPFGGGPRMCPGKEYARLEILVFMHHLVKRFKFEKIIPHE-KIIVNPMPI 460

CYP716AL1 DGSGPAPYTFVPFGGGPRMCPGKEYARLEILVFMHNLVKRFKWEKIIPNE-KIVVDPMPI 465

CYP716A48 EGSGPAPYTFVPFGGGPRMCPGKEYARLEILVFMHHLVKRFKWEKLIPDE-KIVVDPMPI 465

:* ** *::::***********:*:**:***:::::::.::.:: : *.* :::*:*:*

CYP716A80 PGNGLPIRIFPHFGS----- 477

CYP716A1 PDKGLPIRIFPQSGS----- 479

CYP716A110 PAKGLPIRLEPQVCGS---- 482

CYP716A79 PEHGLPVRLFPHPQTAVAGS 485

CYP716A12 PAKDLPIRLYPHKAGS---- 481

CYP716A179 PANDLPIRLYPHDTGS---- 483

CYP716A147 PAKGLPVRLLPHKPGS---- 480

CYP716A75 PEKGLPIRLFSYDAGS---- 489

CYP716A140 PAKGLPIRLYPHKAGS---- 481

CYP716A15 PAKGLPVRLHPHKPGS---- 482

CYP716A83 PAKGLPIRLFPHKAGS---- 483

CYP716A52v2 PANGLPVRLFPHKAGS---- 483

CYP716A244 PASGLPVRLFPHKAGS---- 483

CYP716A44 PANGLPLRLYPHHHNPGS-- 478

CYP716AL1 PEKGLPVRLYPHINAGS--- 482

CYP716A48 PAKGLPIRLYPLNAGS---- 481

* .**:*:

**Fig. S2.** **Multiple sequence alignment of CYP716A proteins.** Protein sequences were aligned using Clustal Omega (www.ebi.ac.uk/Tools/msa/clustalo/) with default parameters. Conserved sequences are highlighted as transmembrane domain (green) predicted using the TMHMM server (<http://www.cbs.dtu.dk/services/TMHMM>); proline cluster that forms a hinge between the transmembrane and cytoplasmic domains (P450 consensus PPxP, purple); A/G-G-X-D/E-T-T/S motif (proton transfer groove, light blue); ExxR motif (blue); PERF motif (red); FxxGxRxCxG motif (heme binding loop, orange). The sequences contained an additional ‘GS’ at the C-terminus but were otherwise identical to the GenBank sequences. The protein sequences were retrieved from GenBank: CYP716A1, NP_198460.1; CYP716A12, CBN88269.1; CYP716A15, BAJ84106; CYP716A44, AK329870.1; CYP716A48, BAP59949.1; CYP716A52v2, AFO63032; CYP716A75, AHF22088.1; CYP716A79, ANY30854.1; CYP716A80, ALR73782.1; CYP716A83, AOG74832.1; CYP716A110, AOG74847.1; CYP716A140, AOG74836.1; CYP716A147, XP_007023618; CYP716A179, BAW34647.1; CYP716A244, APZ88353.1; CYP716AL1, AEX07773.
