# Supplementary material for: A systematic comparison of triterpenoid biosynthetic enzymes for the production of oleanolic acid in Saccharomyces cerevisiae
Source: PLoS One. 2020 May 1;15(5):e0231980. doi: 10.1371/journal.pone.0231980 (PMC7194398; doi:10.1371/journal.pone.0231980)
Supplement: S3 Fig — (A) Total ion chromatogram (TIC) of strains expressing AaBAS, BvBAS and the MD-N1 control strain (expressing no BAS). While both AaBAS and BvBAS have a peak corresponding to β-amyrin, the BvBAS strain has a second unique peak (*) that is potentially a combination of two peaks and which is not present in either the AaBAS or control strains. (B) Mass spectrum at retention time marked by asterix (*) (top). Comparison with a mass spectrum of an authentic lupeol standard (bottom) suggests one of the compounds contained within the peak corresponds to lupeol. (DOCX) [file pone.0231980.s003.docx]

**
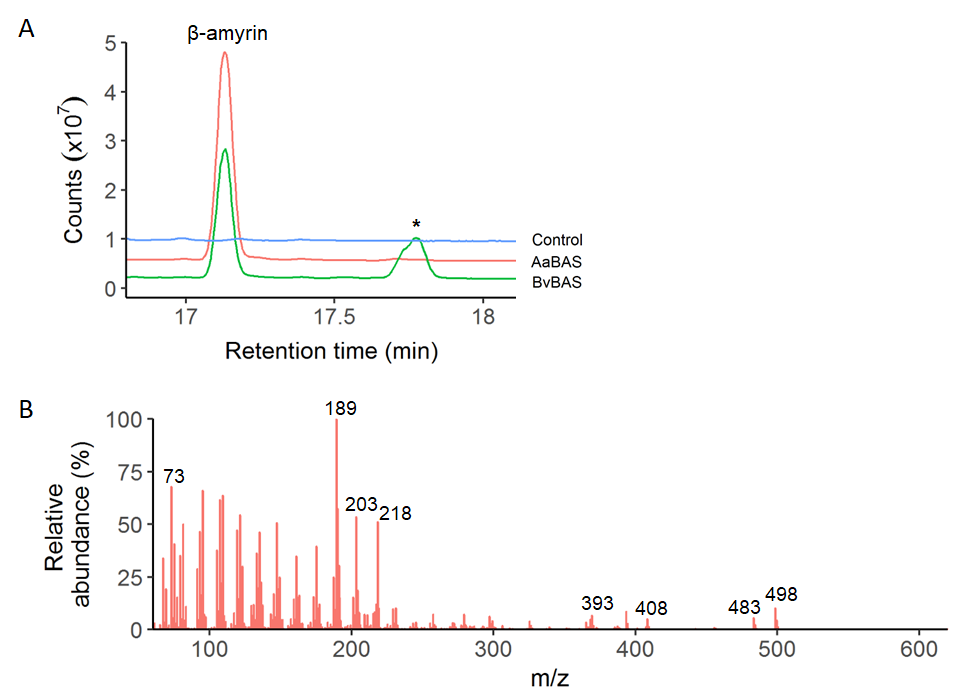
**

**C**


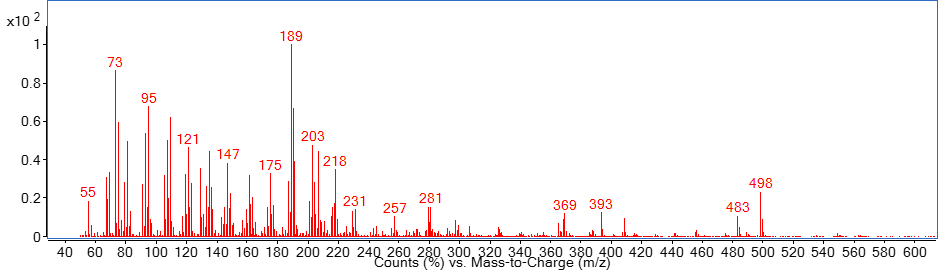


**Fig. S3. BvBAS is a multifunctional OSC producing multiple triterpenes. (A)** Total ion chromatogram (TIC) of strains expressing AaBAS, BvBAS and the MD-N1 control strain (expressing no BAS). While both AaBAS and BvBAS have a peak corresponding to β-amyrin, the BvBAS strain has a second unique peak (*) that is potentially a combination of two peaks and which is not present in either the AaBAS or control strains. **(B)** Mass spectrum at retention time marked by asterix (*) (top). Comparison with a mass spectrum of an authentic lupeol standard (bottom) suggests one of the compounds contained within the peak corresponds to lupeol.
