# Supplementary material for: A systematic comparison of triterpenoid biosynthetic enzymes for the production of oleanolic acid in Saccharomyces cerevisiae
Source: PLoS One. 2020 May 1;15(5):e0231980. doi: 10.1371/journal.pone.0231980 (PMC7194398; doi:10.1371/journal.pone.0231980)
Supplement: S1 Table — (DOCX) [file pone.0231980.s004.docx]

**Supplementary Table S1.** List of enzymes.

| **Enzyme** | **Species** | **Accession number** | | **Reference** |
| --- | --- | --- | --- | --- |
| AsBAS | *Avena strigosa* | CAC84558.1 | (1) | |
| PgBAS1 | *Panax ginseng* | BAA33461.1 | (2) | |
| GgBAS | *Glycyrrhiza glabra* | BAA89815.1 | (3) | |
| LjBAS | *Lotus japonicus* | BAE53429.1 | (4) | |
| EtBAS | *Euphorbium tirucalli* | BAE43642.1 | (5) | |
| PtBAS | *Polygala tenuifolia* | ABL07607.1 | (6) | |
| GhBAS | *Gossypium hirsutum* | XP_016749748.1 | The present study | |
| AaBAS | *Artemisia annua* | ACA13386.1 | (7) | |
| BvBAS | *Barbarea vulgaris* | AFF27505.1 | (8) | |
| CqBAS1 | *Chenopodium quinoa* | ANY30852.1 | (9) | |
| MtBAS | *Medicagoa truncatula* | CAD23247.1 | (10) | |
| SlBAS | *Solanum lycopersicum* | ADU52574.1 | (11) | |
| CYP716A12 | *Medicago truncatula* | CBN88269.1 | (12) | |
| CYP716A15 | *Vitis vinifera* | BAJ84106 | (12) | |
| CYP716AL1 | *Catharanthus roseus* | AEX07773 | (13) | |
| CYP716A52v2 | *Panax ginseng* | AFO63032 | (14,15) | |
| CYP716A147 | *Theobroma cacao* | XP_007023618 | The present study | |
| CYP716A48 | *Olea europaea* | BAP59949.1 | The present study | |
| CYP716A44 | *Solanum lycopersicum* | AK329870.1 | (16) | |
| CYP716A75 | *Maesa lanceolata* | AHF22088.1 | (17) | |
| CYP716A79 | *Chenopodium quinoa* | ANY30854.1 | (9) | |
| CYP716A80 | *Barbarea vulgaris* | ALR73782.1 | (18) | |
| CYP716A83 | *Centella asiatica* | AOG74832.1 | (19,20) | |
| CYP716A110 | *Aquilegia coerulea* | AOG74847.1 | (19) | |
| CYP716A140 | *Platycodon grandiflorus* | AOG74836.1 | (19) | |
| CYP716A179 | *Glycyrrhiza uralensis* | BAW34647.1 | (21) | |
| CYP716A244 | *Eleutherococcus senticosus* | APZ88353.1 | (22) | |
| CYP716A1 | *Arabidopsis thaliana* | NP_198460.1 | (23) | |
| ATR2 | *Arabidopsis thaliana* | NP_849472.2 | (24) | |

**References**

1. Haralampidis K, Bryan G, Qi X, Papadopoulou K, Bakht S, Melton R, et al. A new class of oxidosqualene cyclases directs synthesis of antimicrobial phytoprotectants in monocots. Proc Natl Acad Sci. 2001;98(23):13431–6.

2. Kushiro T, Shibuya M, Ebizuka Y. β-Amyrin synthase - Cloning of oxidosqualene cyclase that catalyzes the formation of the most popular triterpene among higher plants. Eur J Biochem. 1998;256:238–44.

3. Hayashi H, Huang P, Kirakosyan A, Inoue K, Hiraoka N, Ikeshiro Y, et al. Cloning and Characterization of a cDNA Encoding β-Amyrin Synthase Involved in Glycyrrhizin and Soyasaponin Biosyntheses in Licorice. Biol Pharm Bull. 2001;24(8):912–6.

4. Iturbe-ormaetxe I, Haralampidis K, Papadopoulou K, Osbourn AE. Molecular cloning and characterization of triterpene synthases from Medicago truncatula and Lotus japonicus. Plant Mol Biol. 2003;51:731–43.

5. Kajikawa M, Yamato KT, Fukuzawa H, Sakai Y. Cloning and characterization of a cDNA encoding β-amyrin synthase from petroleum plant Euphorbia tirucalli L. Phytochemistry. 2005;66:1759–66.

6. Jin ML, Lee DY, Um Y, Lee JH, Park CG, Jetter R, et al. Isolation and characterization of an oxidosqualene cyclase gene encoding a β-amyrin synthase involved in Polygala tenuifolia Willd. saponin biosynthesis. Plant Cell Rep. 2014;33:511–9.

7. Kirby J, Romanini DW, Paradise EM, Keasling JD. Engineering triterpene production in Saccharomyces cerevisiae – β-amyrin synthase from Artemisia annua. FEBS J. 2008;275:1852–9.

8. Wei X, Zhang X, Wu Q, Wang H, Shen D, Qiu Y, et al. Cloning, Characterization and Real-time RT-PCR Analysis of a Key Gene β-Amyrin synthase for Saponin Biosynthesis in Barbarea vulgaris. Acta Hortic Sin. 2012;39:923–30.

9. Fiallos-Jurado J, Pollier J, Moses T, Arendt P, Barriga-medina N, Morillo E, et al. Saponin determination, expression analysis and functional characterization of saponin biosynthetic genes in Chenopodium quinoa leaves. Plant Sci. 2016;250:188–97.

10. Suzuki H, Achnine L, Xu R, Matsuda SPT, Dixon RA. A genomics approach to the early stages of triterpene saponin biosynthesis in Medicago truncatula. Plant J. 2002;32:1033–48.

11. Wang Z, Guhling O, Yao R, Li F, Yeats TH, Rose JKC, et al. Two Oxidosqualene Cyclases Responsible for Biosynthesis of Tomato Fruit Cuticular Triterpenoids. Plant Physiol. 2011;155:540–52.

12. Fukushima EO, Seki H, Ohyama K, Ono E, Umemoto N, Mizutani M, et al. CYP716A Subfamily Members are Multifunctional Oxidases in Triterpenoid Biosynthesis. Plant Cell Physiol. 2011;52(12):2050–61.

13. Huang L, Li J, Ye H, Li C, Wang H, Liu B, et al. Molecular characterization of the pentacyclic triterpenoid biosynthetic pathway in Catharanthus roseus. Planta. 2012;236:1571–81.

14. Han J, Hwang H, Choi S, Kim H, Choi Y. Cytochrome P450 CYP716A53v2 Catalyzes the Formation of Protopanaxatriol from Protopanaxadiol During Ginsenoside Biosynthesis in Panax Ginseng. Plant Cell Physiol. 2012;53(9):1535–45.

15. Han J, Kim M, Ban Y, Hwang H, Choi Y. The Involvement of β-Amyrin 28-Oxidase (CYP716A52v2) in Oleanane-Type Ginsenoside Biosynthesis in Panax ginseng. Plant Cell Physiol. 2013;54(12):2034–46.

16. Yasumoto S, Seki H, Shimizu Y, Fukushima EO, Muranaka T. Functional Characterization of CYP716 Family P450 Enzymes in Triterpenoid Biosynthesis in Tomato. Front Plant Sci. 2017;8.

17. Moses T, Pollier J, Faizal A, Apers S, Pieters L, Thevelein JM, et al. Unraveling the Triterpenoid Saponin Biosynthesis of the African Shrub Maesa lanceolata. Mol Plant. 2015;8:122–35.

18. Khakimov B, Kuzina V, Erthmann PØ, Fukushima EO, Augustin J, Olsen CE, et al. Identification and genome organization of saponin pathway genes from a wild crucifer, and their use for transient production of saponins in Nicotiana benthamiana. Plant J. 2015;84(3):478–90.

19. Miettinen K, Pollier J, Buyst D, Arendt P, Csuk R, Sommerwerk S, et al. The ancient CYP716 family is a major contributor to the diversification of eudicot triterpenoid biosynthesis. Nat Commun. 2017;8:14153.

20. Kim OT, Um Y, Jin ML, Kim JU, Hegebarth D, Busta L, et al. A Novel Multifunctional C-23 Oxidase, CYP714E19, is Involved in Asiaticoside Biosynthesis. Plant Cell Physiol. 2018;0:1–14.

21. Tamura K, Seki H, Suzuki H, Kojoma M, Saito K, Muranaka T. CYP716A179 functions as a triterpene C-28 oxidase in tissue-cultured stolons of Glycyrrhiza uralensis. Plant Cell Rep. 2017;36(3):437–45.

22. Jo H, Han JY, Hwang H, Choi YE. β-Amyrin synthase (EsBAS) and β-amyrin 28-oxidase (CYP716A244) in oleanane-type triterpene saponin biosynthesis in Eleutherococcus senticosus. Phytochemistry. 2017;135:53–63.

23. Yasumoto S, Fukushima EO, Seki H, Muranaka T. Novel triterpene oxidizing activity of Arabidopsis thaliana CYP716A subfamily enzymes. FEBS Lett. 2016;590:533–40.

24. Urban P, Mignotte C, Kazmaier M, Delorme F, Pompon D. Cloning, yeast expression, and characterization of the coupling of two distantly related Arabidopsis thaliana NADPH-cytochrome P450 reductases with P450 CYP73A5. J Biol Chem. 1997;272(31):19176–86.
